# Supplementary material for: The Chloranthus sessilifolius genome provides insight into early diversification of angiosperms
Source: Nat Commun. 2021 Nov 26;12:6929. doi: 10.1038/s41467-021-26931-3 (PMC8626421; doi:10.1038/s41467-021-26931-3)
Supplement: Supplementary file 1 — Supplementary Information [file 41467_2021_26931_MOESM1_ESM.pdf]

The *Chloranthus sessilifolius* genome provides insight into early diversification of angiosperms

Ma *et al.*

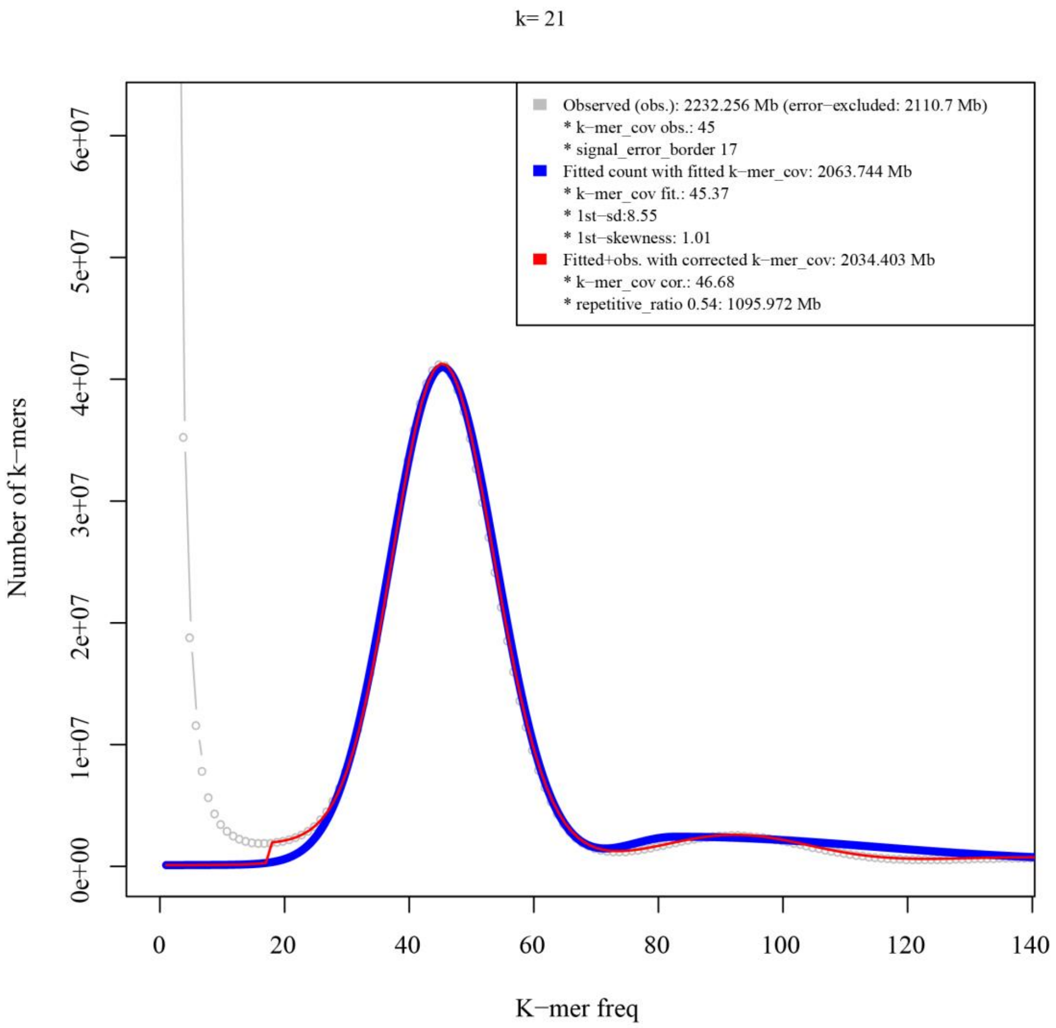

Supplementary Figure 1. Genome size estimation by findGSE. K-mer size was set as 17 and the default parameters were used.

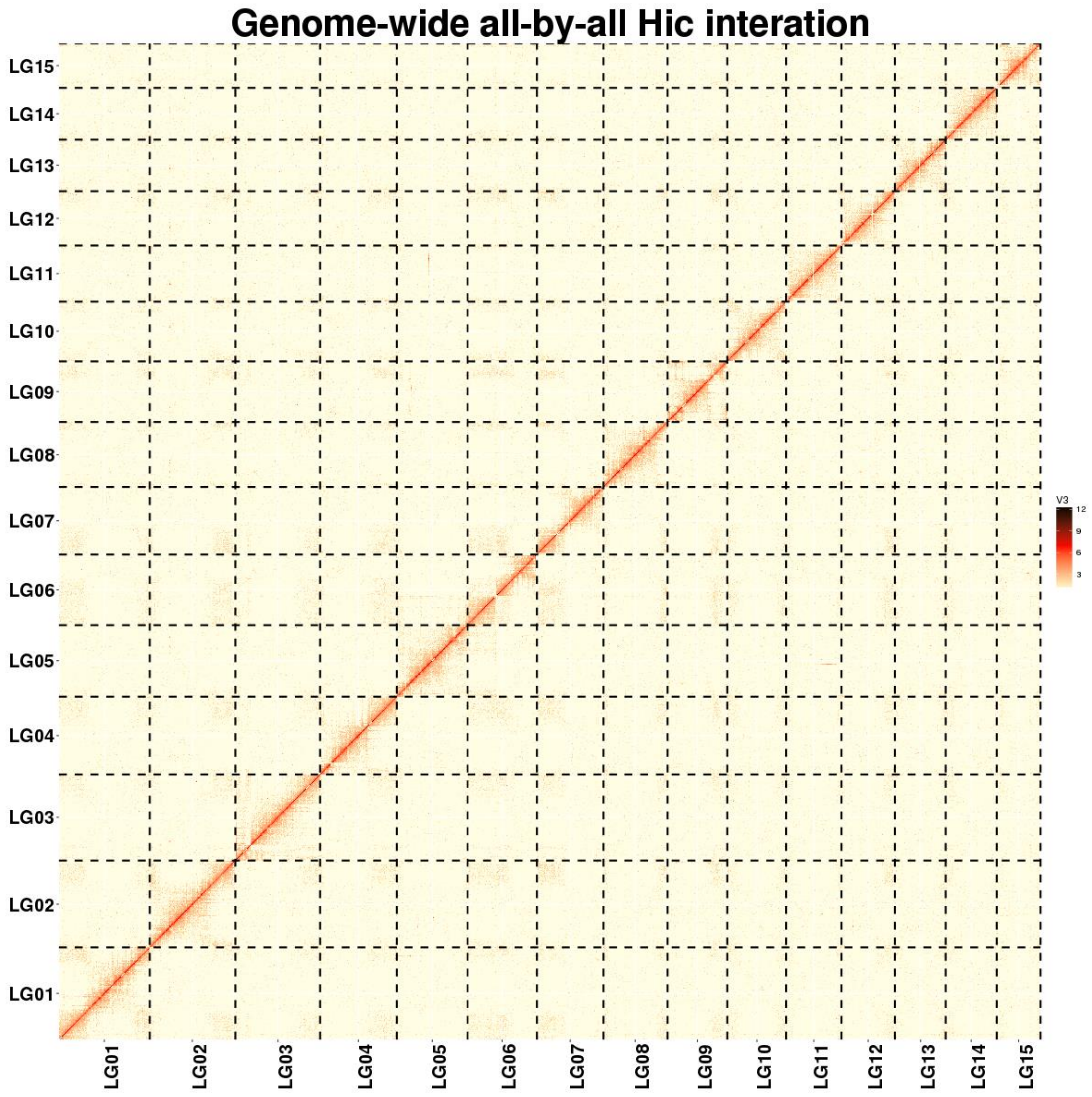

Supplementary Figure 2. Chromosome-level assembly of the *Chloranthus sessilifolius* genome using Hi-C technology.

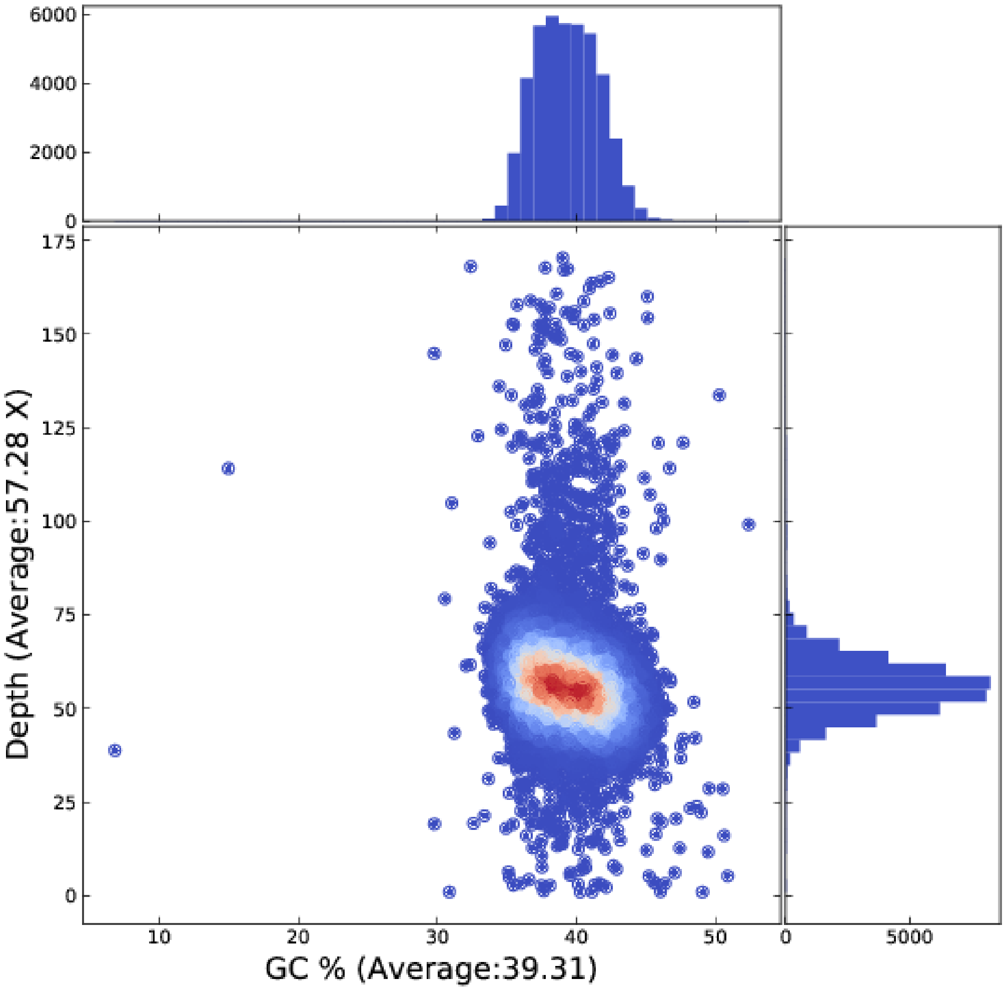

Supplementary Figure 3. The genome-wide GC depth distribution of *Chloranthus sessilifolius*. For the genome-wide analysis, the 500 bp non-overlapping sliding windows were generated.

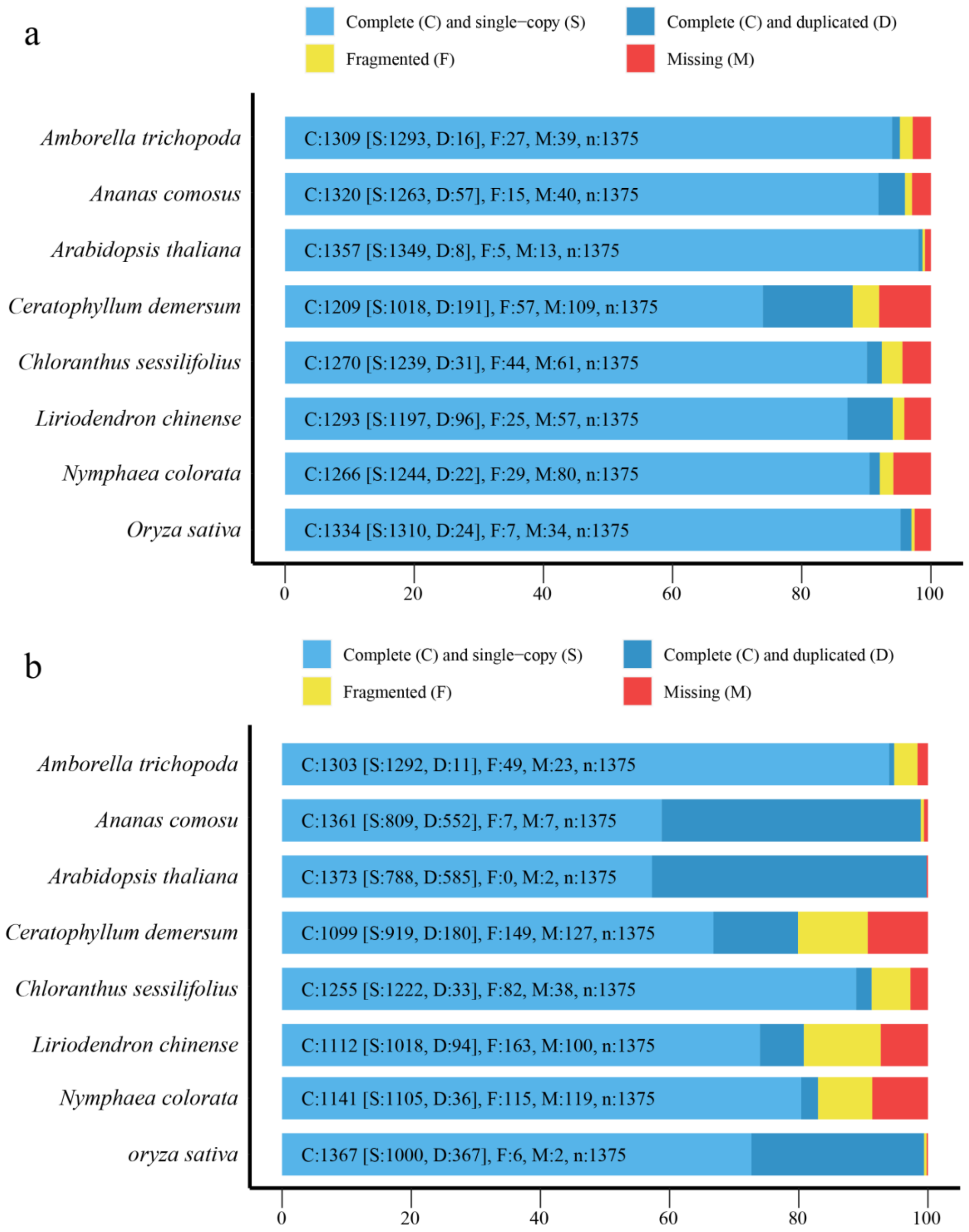

Supplementary Figure 4. The BUSCO results of *C. sessilifolius* and the other representative species using the genome assemblies (a) and proteins (b).

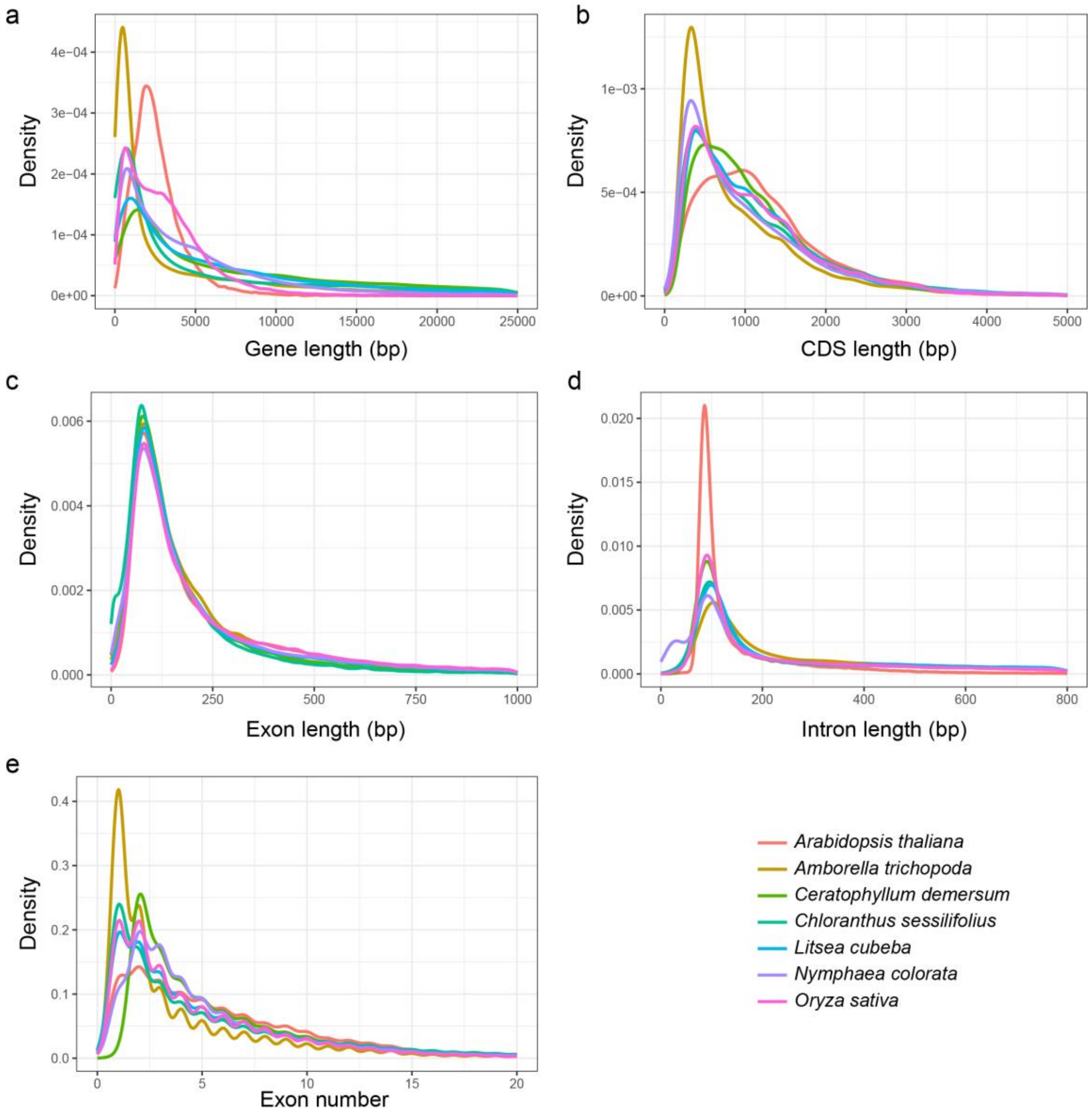

Supplementary Figure 5. **Comparison of gene structure characteristics in *Chloranthus sessilifolius* to that in other six plants**. (a) gene length; (b) CDS length; (c) exon length; (d) intron length; (e) exon number.

a

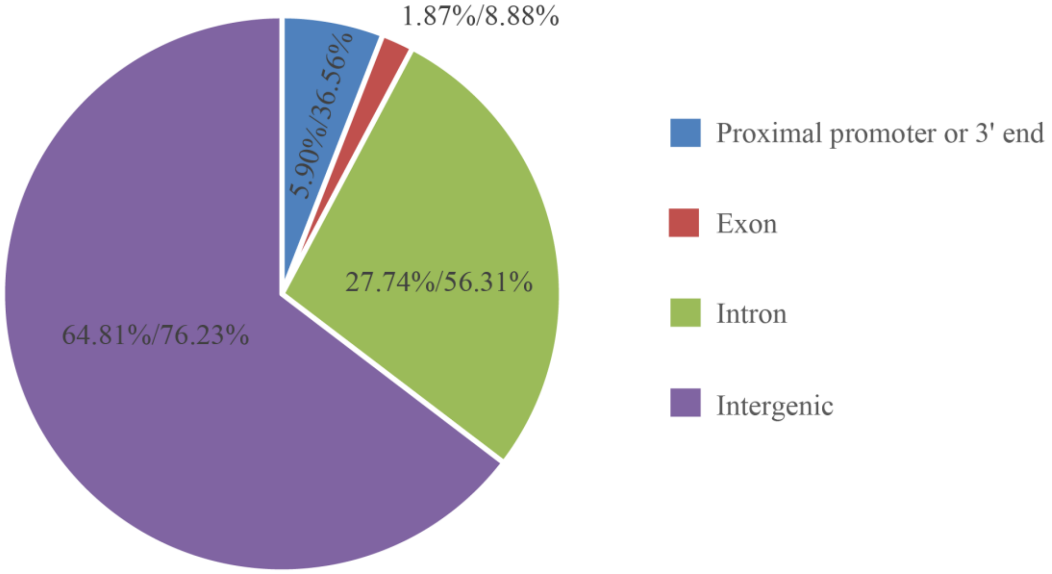

Supplementary Figure 6. TE distribution across the *Chloranthus* genome. (a) The pie graph demonstrates four separate genomic regions, i.e., 2,000 bp upstream or downstream of the gene (blue), exon (red), intron (green) and intergenic regions (pink). Two aspects of genomic composition were shown using the '/' as a separator, which the former indicates the genomic regions accounted for the proportion of the whole genome and the latter represents the proportion of the TE region in the corresponding genomic region.

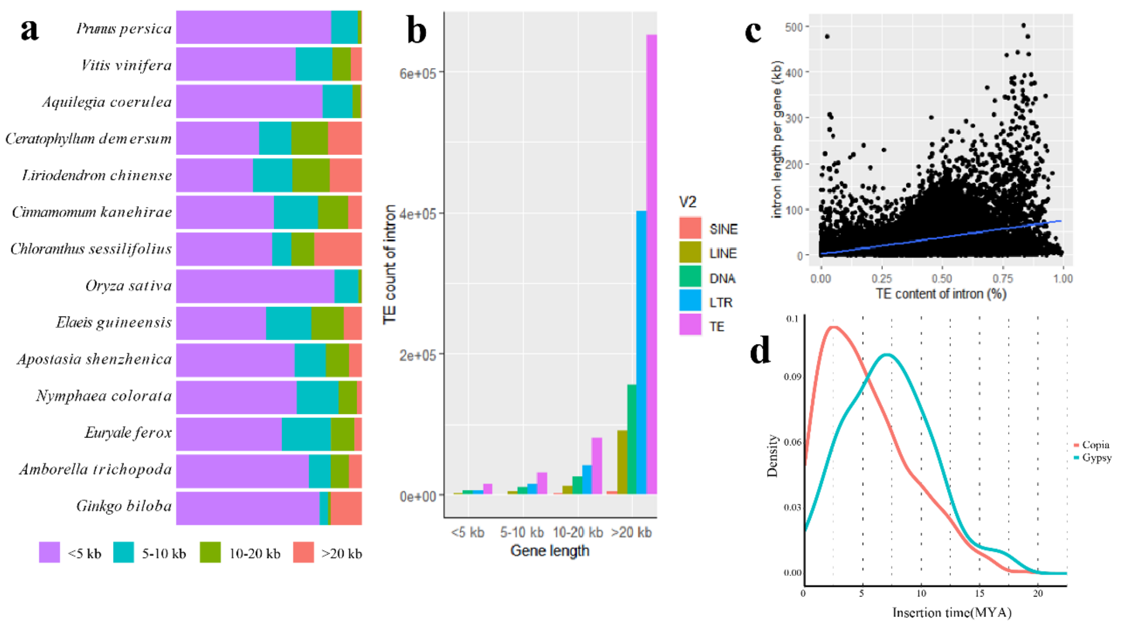

Supplementary Figure 7. Characterization of genes and TEs in *Chloranthus sessilifolius*. (a) The percentage of genes with different length ranges is shown for each species. (b) The content of different types of TE in different length genes in *Chloranthus sessilifolius*. (c) The number of TE in introns is positively correlated with the length of introns in each gene. (d) Insertion times of LTR retrotransposons.

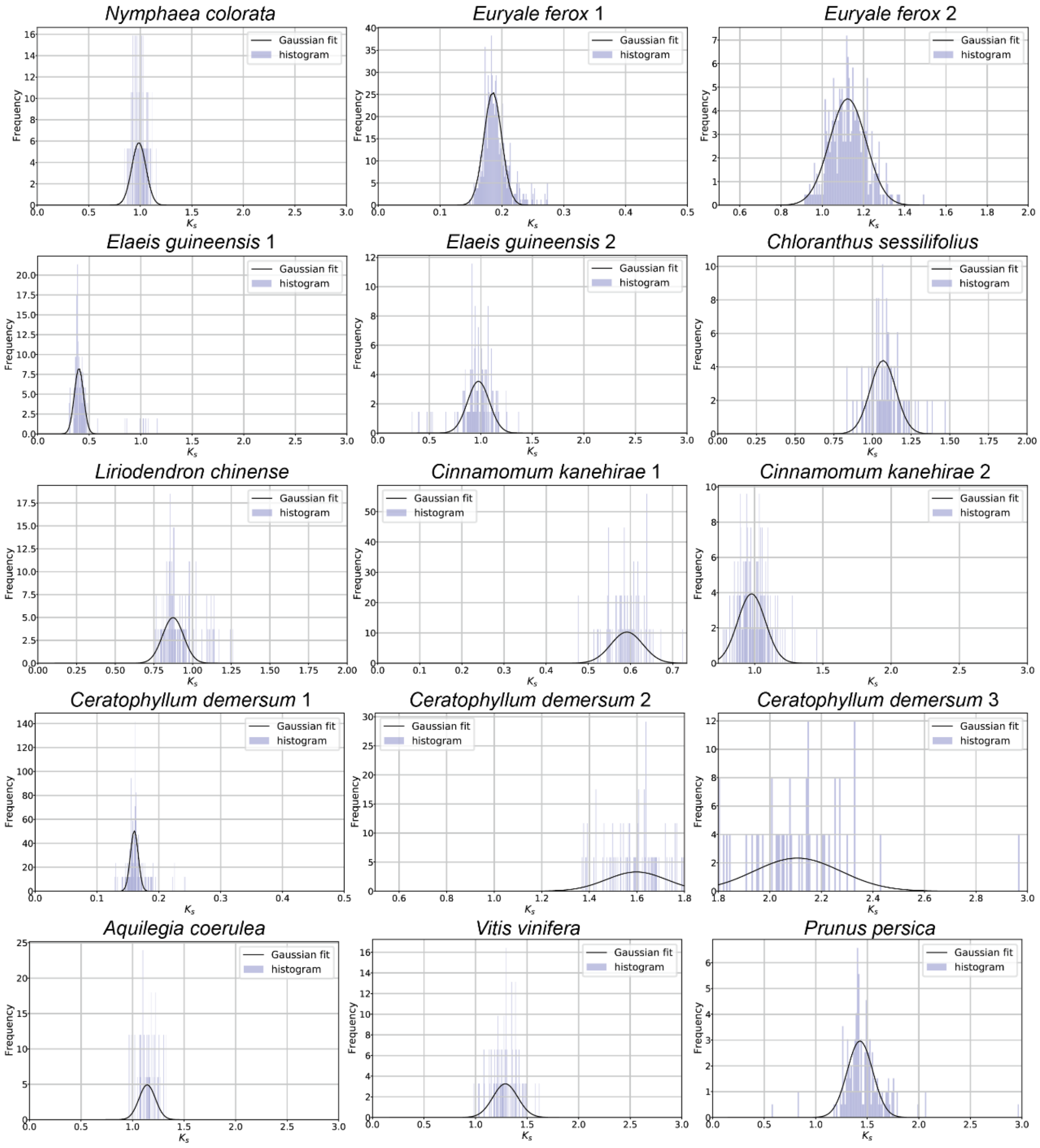

Supplementary Figure 8. Distribution of *Ks* of intragenomic syntenic blocks of each species. The histogram showed the raw *Ks* distribution and the black curve represent the Gaussian fit of the raw *Ks*. All the peaks correspond to Figure 2a in the main text. Source data are provided as a Source Data file.

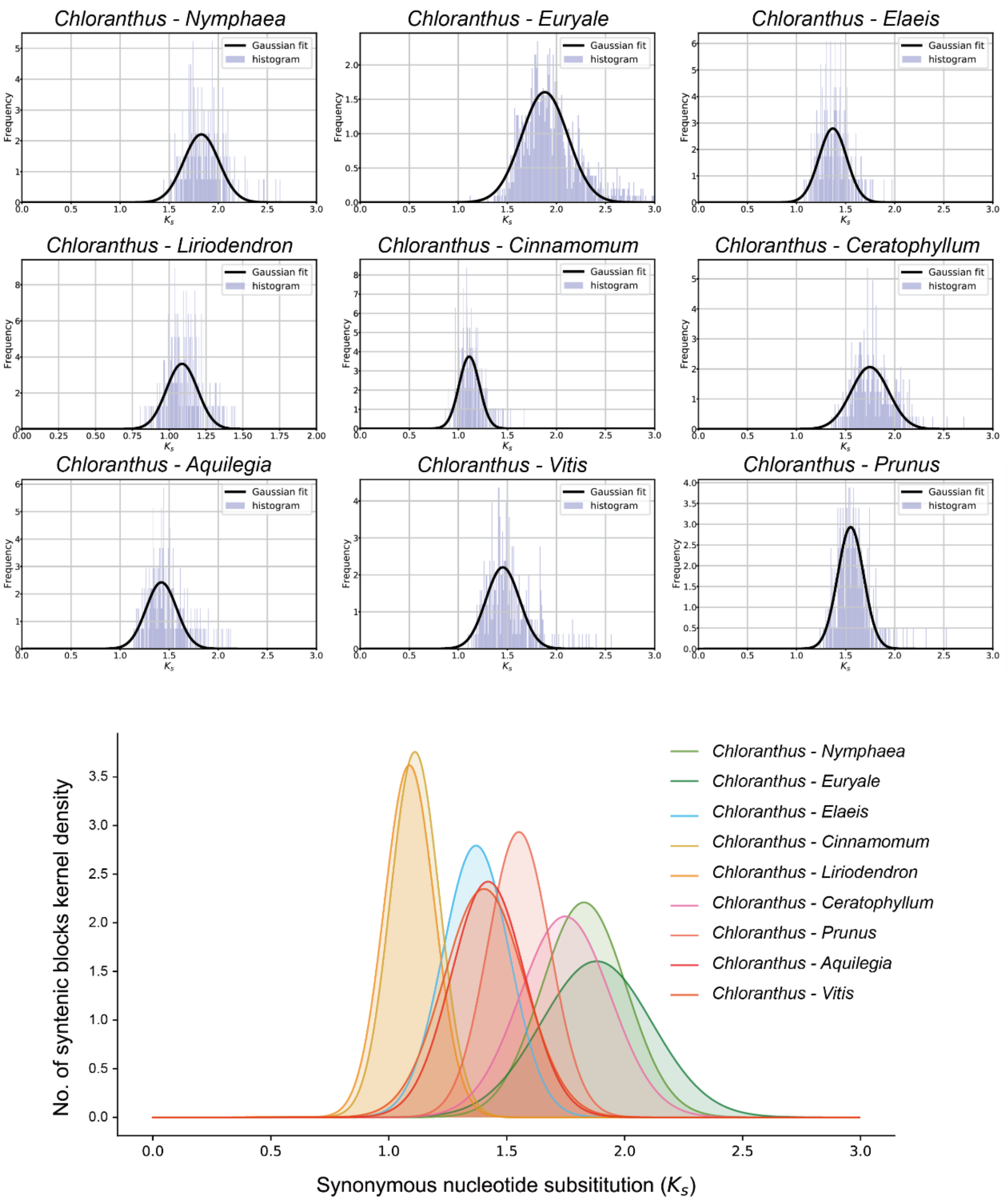

Supplementary Figure 9. Distribution of *Ks* of intergenomic syntenic blocks between *Chloranthus* and other species. The histogram showed the raw *Ks* distribution and the black curve represent the Gaussian fit of the raw *Ks* and this also showed in the bottom.

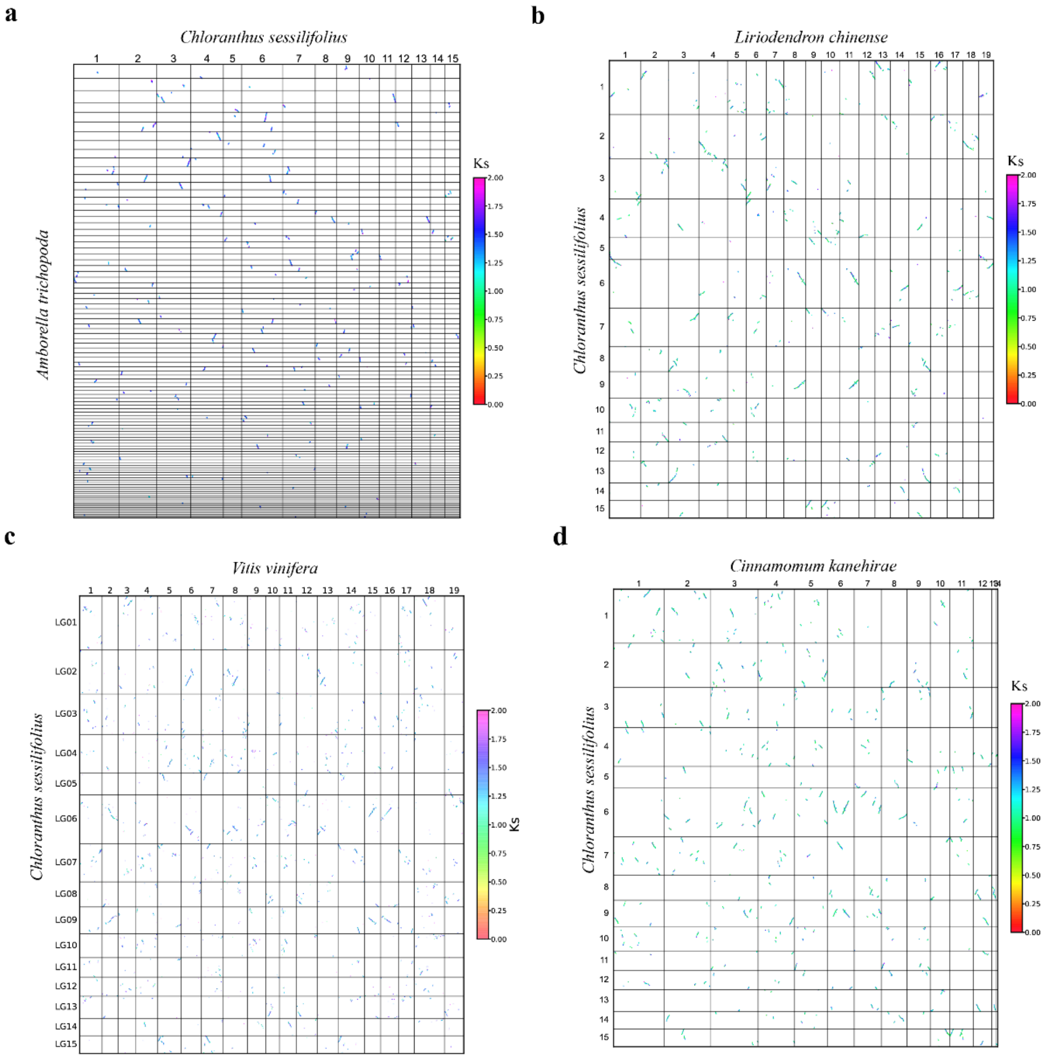

Supplementary Figure 10. Syntenic block dotplot between *Chloranthus sessilifolius* and *Amborella trichopoda* (a), *Chloranthus sessilifolius* and *Vitis vinifera* (b), *Chloranthus sessilifolius* and *Liriodendron chinense* (c), and *Chloranthus sessilifolius* and *Cinnamomum kanehirae* (d).

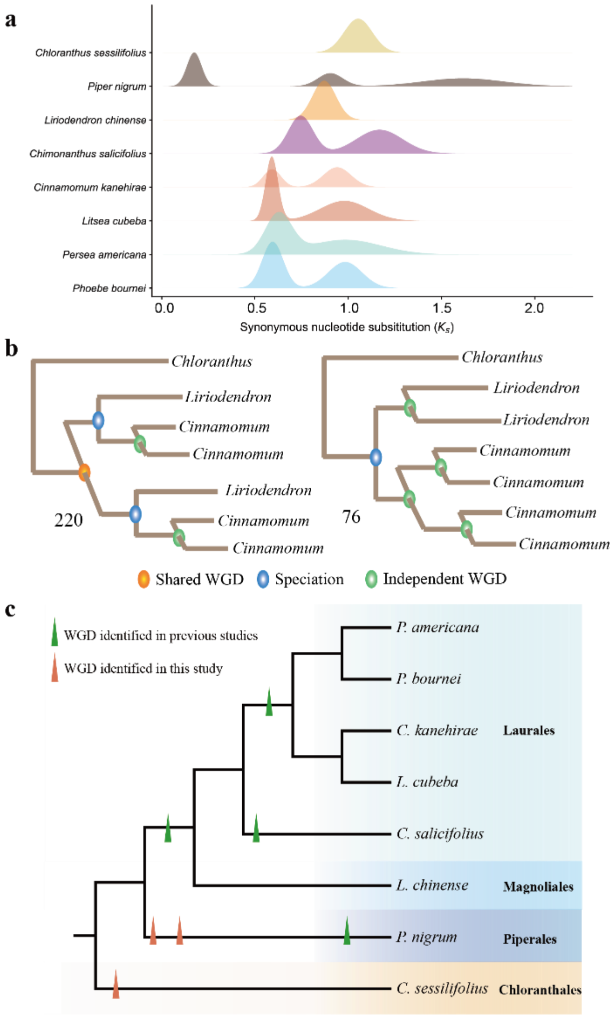

Supplementary Figure 11. Polyploidization history within magnoliids. a, The *Ks* distribution of intragenomic synteny blocks. b, Two possible Liriodendron and Cinnamomum speciation scenarios, the number in the lower-left corner of each tree represents the number of gene trees supporting the topology. c, Schematic diagram of the WGD events in the history of magnoliids. The shared or independent WGD events were both based on the collinear gene tree statistics as shown in b.

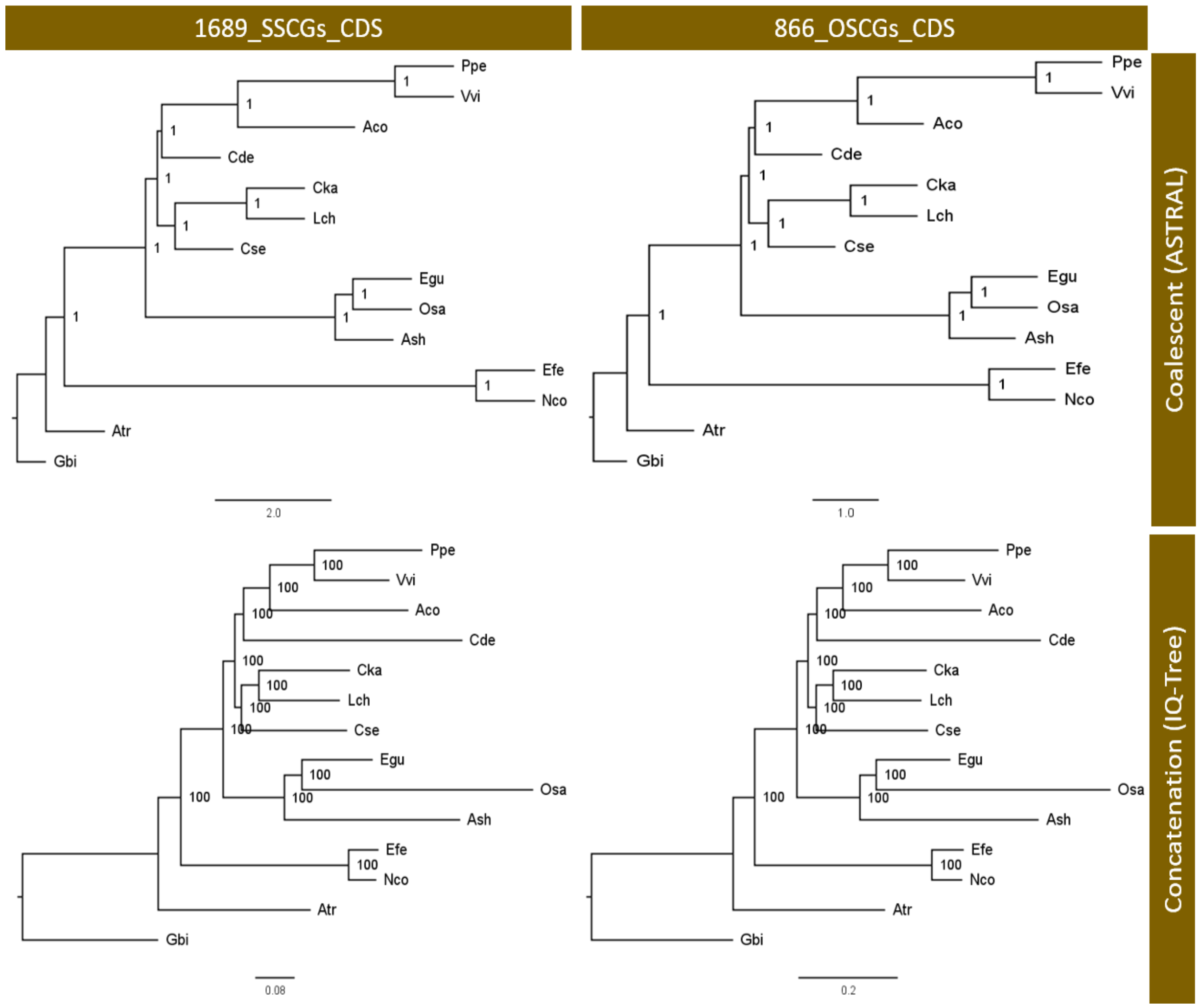

Supplementary Figure 12. ASTRAL- and concatenated-based phylogenetic trees using nucleotide sequence. The corresponding relationship between abbreviations and full names are listing as follow: Aco - *A. coerulea,* Atr - *A. trichopoda,* Ash – *A. shenzhenica,* Cde - *C. demersum,* Cka - *C. kanehirae,* Cse - *C. sessilifolius,* Efe - *E. ferox,* Egu – *E. guineensis* , Gbi - *G. biloba,* Lch - *L. chinense,* Nco – *N. colorata,* Osa - *O. sativa* , Pab - *P. abies,* Ppe - *P. persica,* Vvi - *V. vinifera*. Source data are provided as a Source Data file.

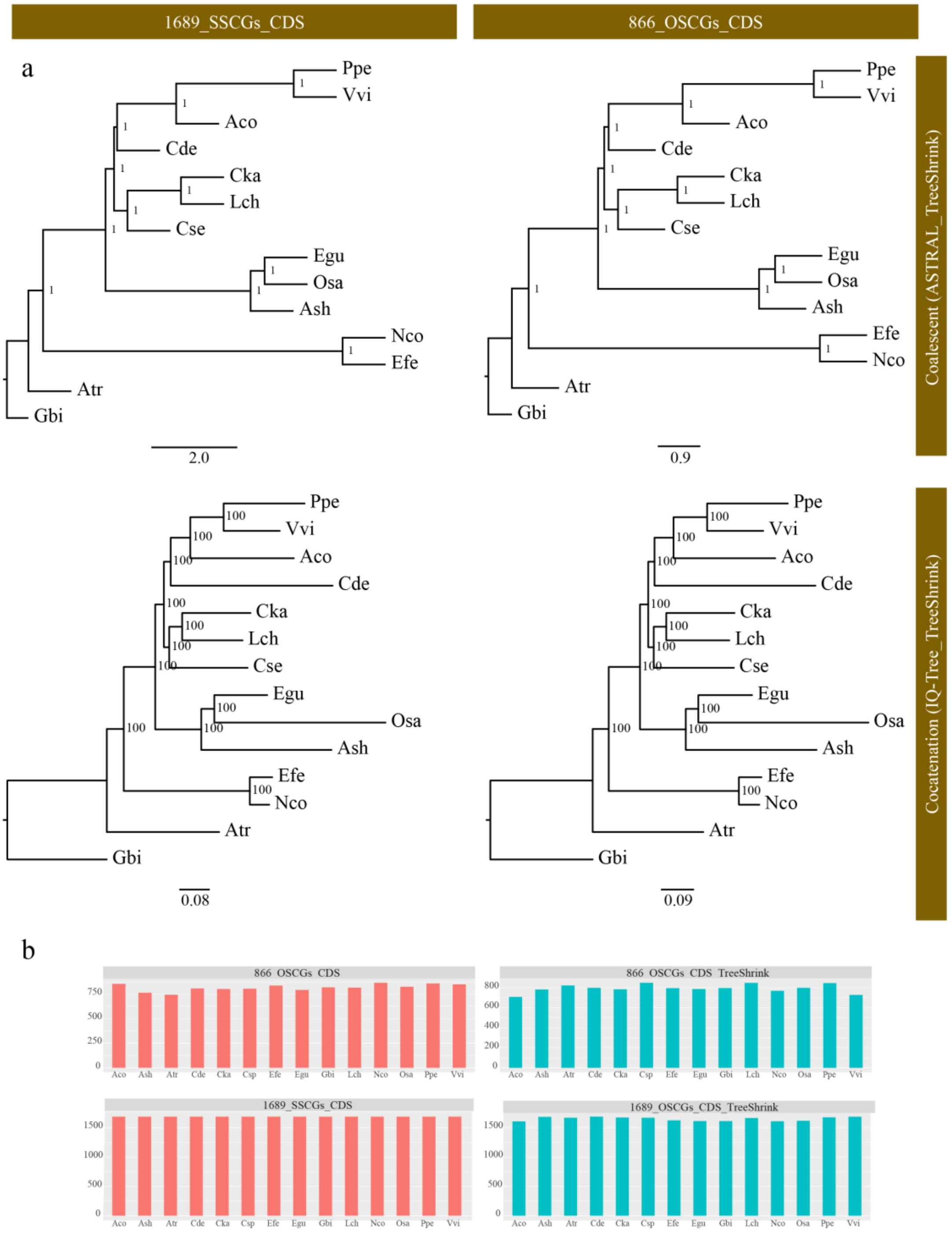

Supplementary Figure 13. TreeShrink analysis for reducing the influence of long-branch attraction. (a) Coalescent and concatenation trees after the TreeShrink filtering. (b) The distribution of species retaining of each dataset.

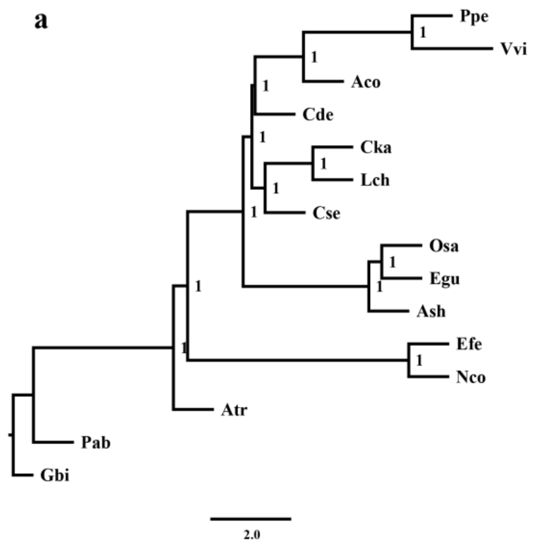

Supplementary Figure 14. Phylogenetic relationships inferred by low-copy orthogroups (2,097 gene families). The two tree topologies were constructed using the STAG (a) and ASTRAL-pro (b), respectively based on the nucleotide sequence. The corresponding relationship between abbreviations and full names are listed as follow: Aco - *A. coerulea,* Atr - *A. trichopoda,* Ash – *A. shenzhenica,* Cde - *C. demersum,* Cka - *C. kanehirae,* Cse - *C. sessilifolius,* Efe - *E. ferox,* Egu – *E. guineensis* , Gbi - *G. biloba,* Lch - *L. chinense,* Nco – *N. colorata,* Osa - *O. sativa* , Pab - *P. abies,* Ppe - *P. persica,* Vvi - *V. vinifera*. Source data are provided as a Source Data file.

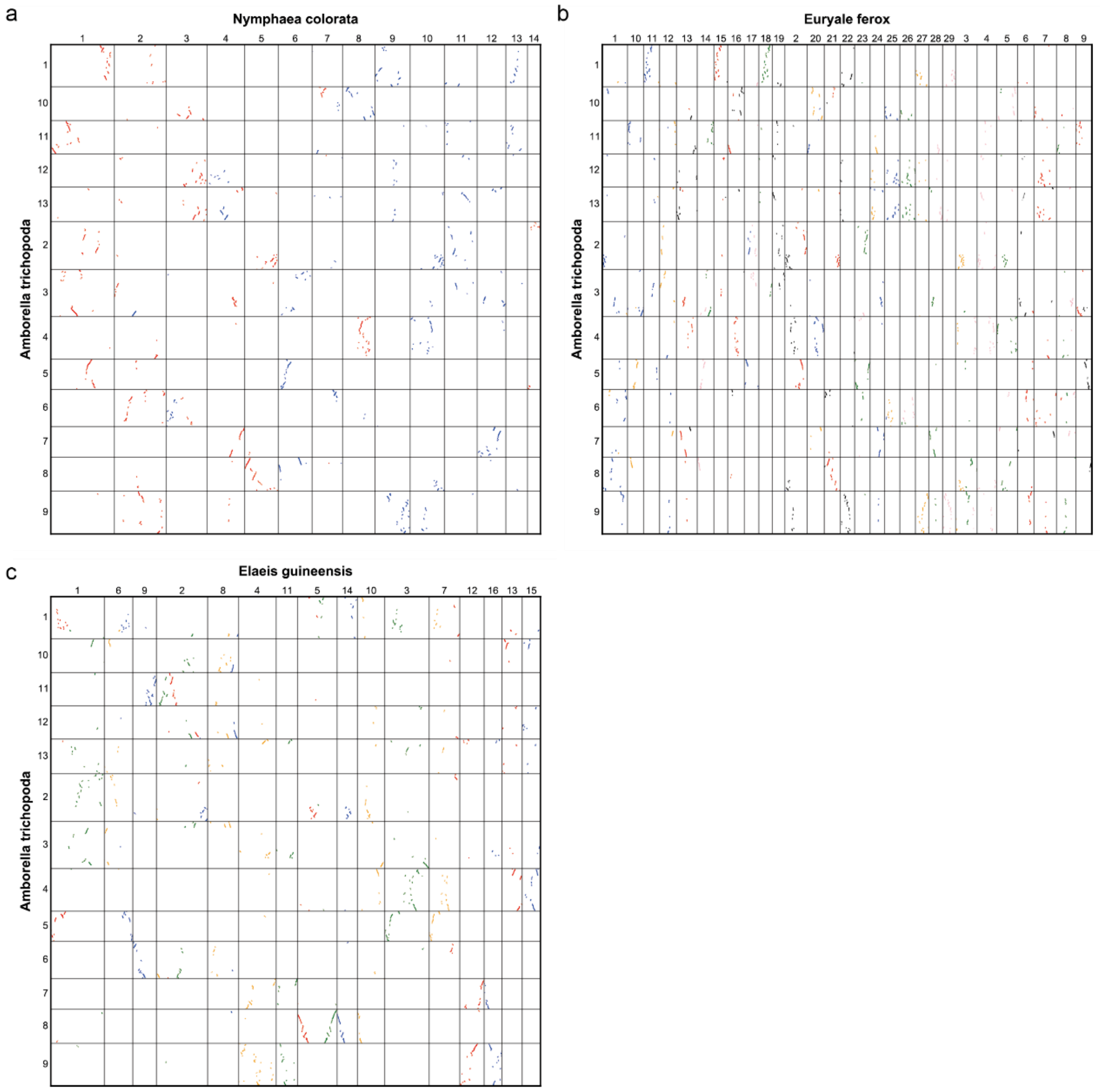

Supplementary Figure 15. Collinear gene extraction between *Amborella* and *Nymphaea* (a), *Amborella* and *Euryale* (b), and *Amborella* and *Elaeis* (c). Different colors represent the different candidate haplotypes of the polyploidization assigned by WGDI.

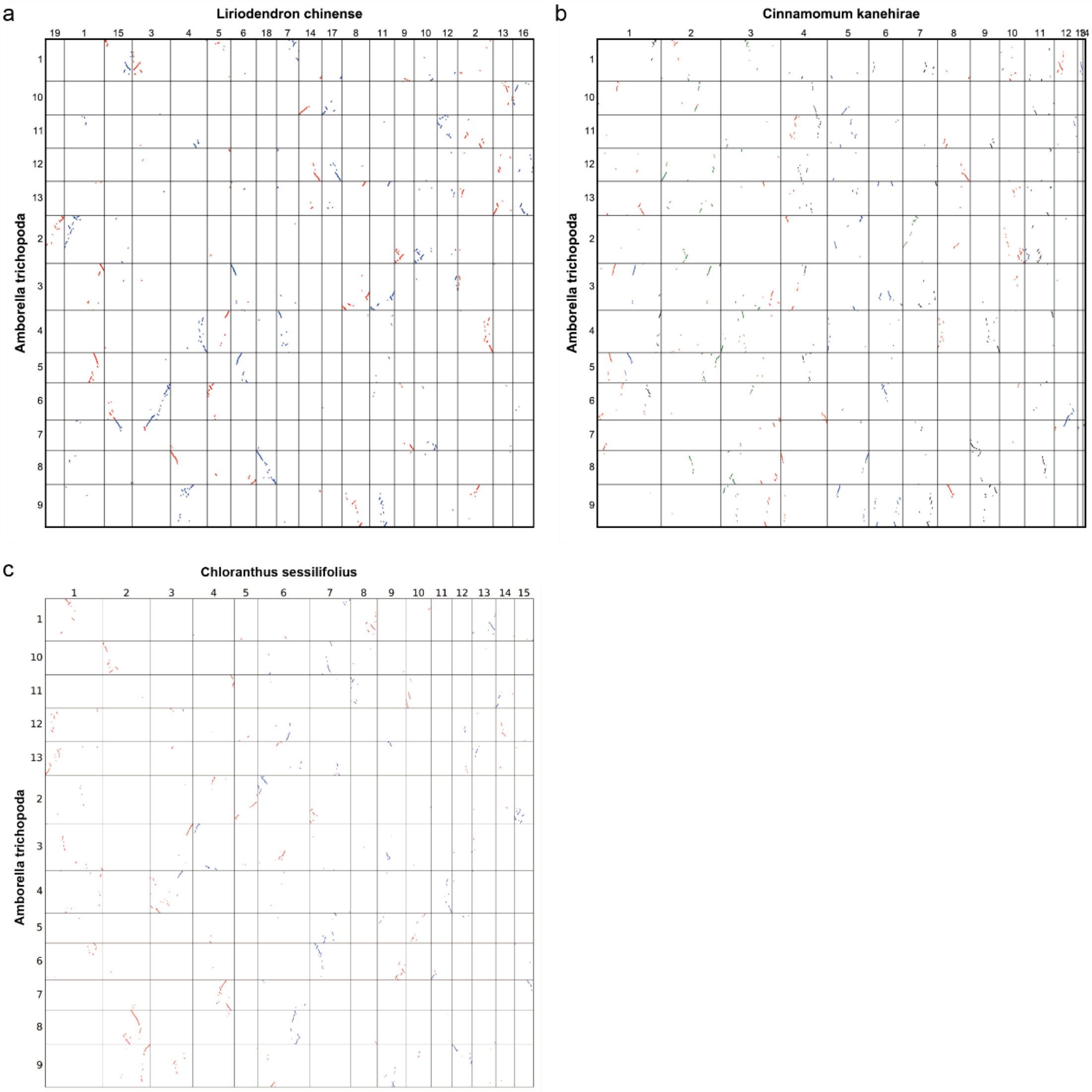

Supplementary Figure 16. Collinear gene extraction between *Amborella* and *Liriodendron* (a), *Amborella* and *Cinnamomum* (b), and *Amborella* and *Chloranthus* (c). Different colors represent the different candidate haplotypes of the polyploidization assigned by WGDI.

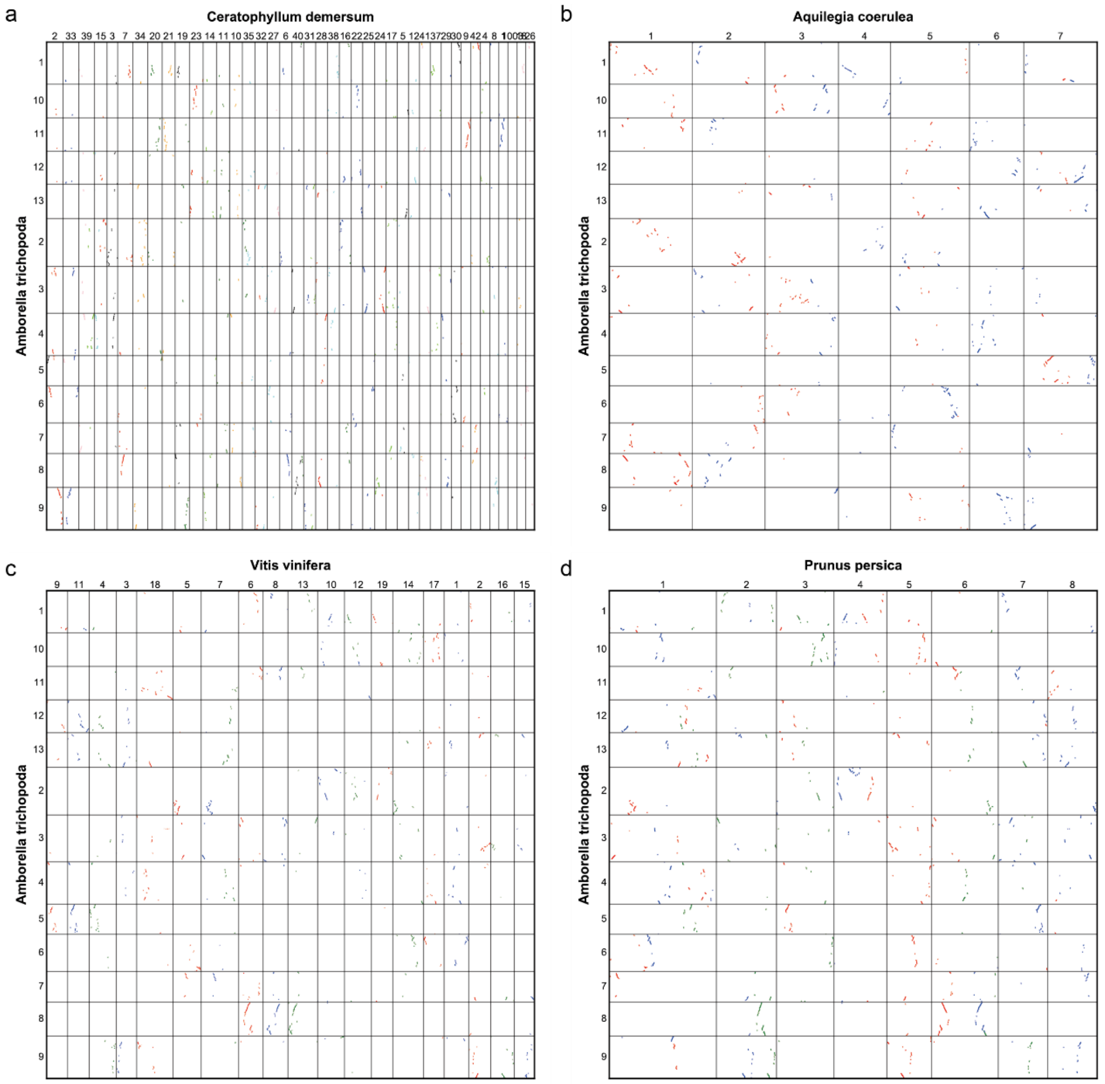

Supplementary Figure 17. Collinear gene extraction between *Amborella* and *Ceratophyllum* (a), *Amborella* and *Aquilegia* (b), *Amborella* and *Vitis* (c), and *Amborella* and *Prunus* (d). Different colors represent the different candidate haplotypes of the polyploidization assigned by WGDI.

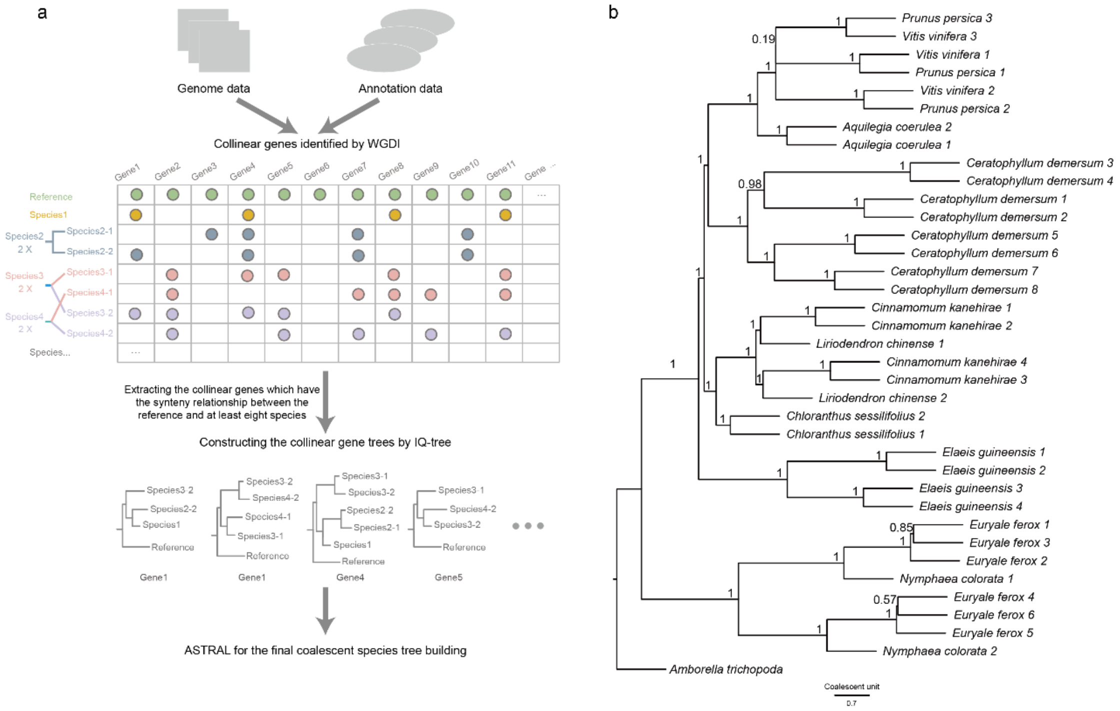

Supplementary Figure 18. Synteny-based species tree constructing among the 11 species. (a) A pipeline of synteny-based species trees constructing. Briefly, if a species had polyploidization, the collinear genes within this species would be assigned into different sets based on the intragenomic synteny information and intergenomic synteny information. (b) Astral tree of the 11 species. The value of each node represents the posterior probability. The internal branch length means the coalescent unit. As ASTRAL couldn’t calculate the branch length for terminal branches, we just set them to 1 only for displaying. Source data are provided as a Source Data file.

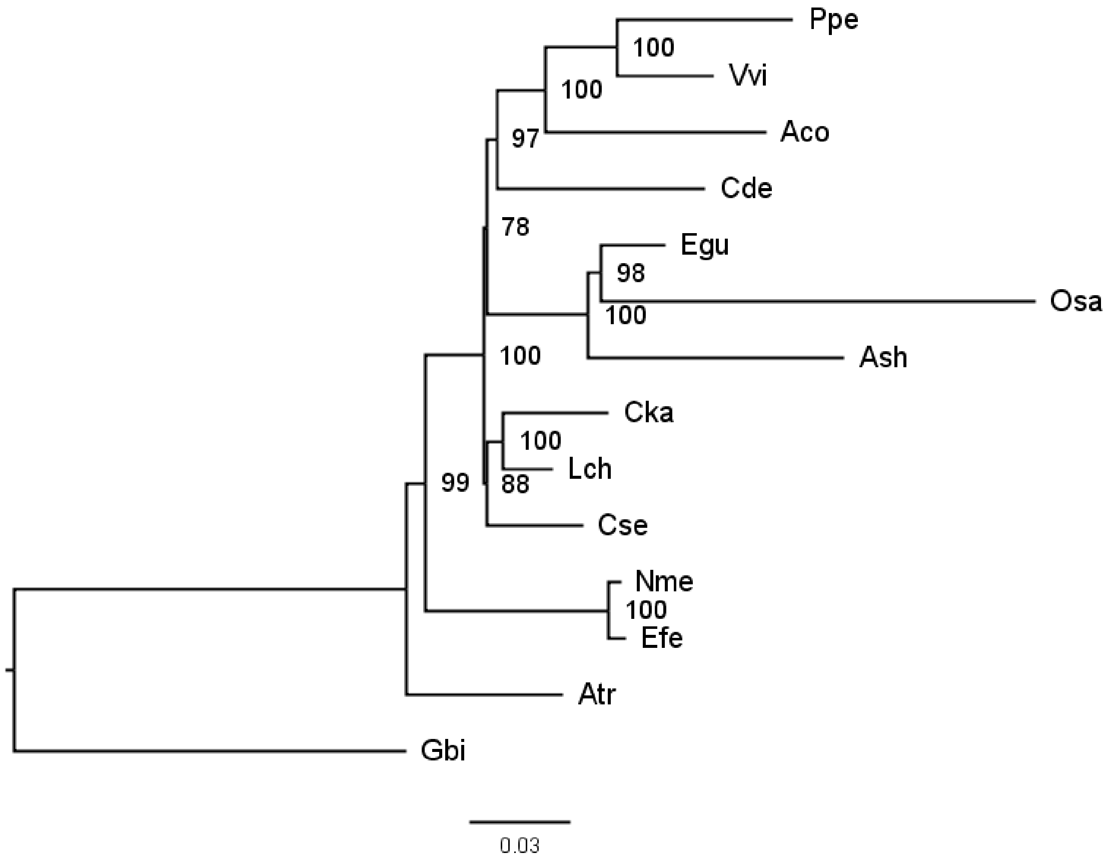

Supplementary Figure 19. The Maximum likelihood (ML) tree is inferred with 80 chloroplast genes from the 14 species based on the concatenated nucleotide sequence. The corresponding relationship between abbreviations and full names are listed as follow: Aco - *A. coerulea,* Atr - *A. trichopoda,* Ash – *A. shenzhenica,* Cde - *C. demersum,* Cka - *C. kanehirae,* Cse - *C. sessilifolius,* Efe - *E. ferox,* Egu – *E. guineensis* , Gbi - *G. biloba,* Lch - *L. chinense,* Nco – *N. colorata,* Osa - *O. sativa* , Pab - *P. abies,* Ppe - *P. persica,* Vvi - *V. vinifera*. Source data are provided as a Source Data file.

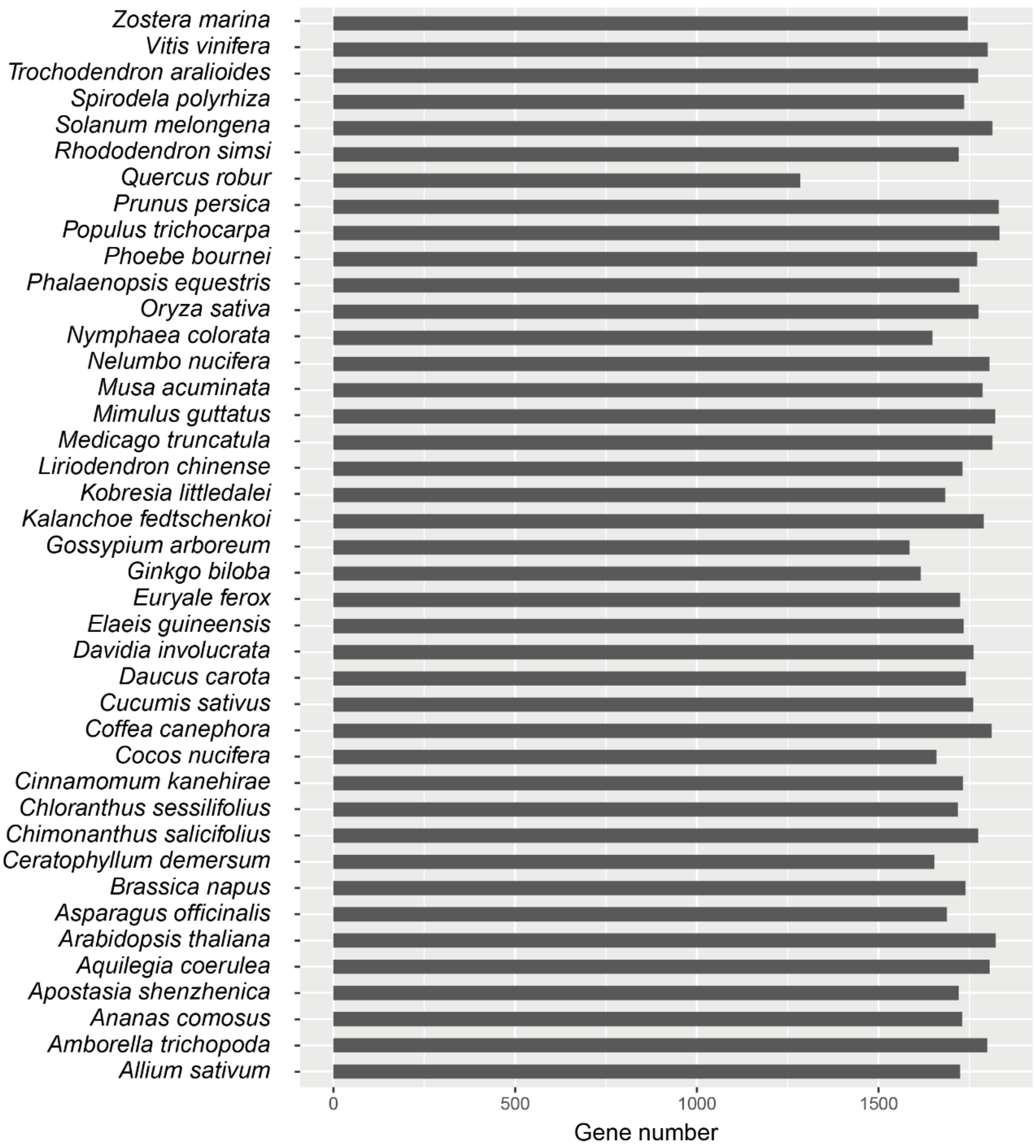

Supplementary Figure 20. The gene number of each species that identified in the 'mostly' single-copy orthologous across the 42 species. Source data are provided as a Source Data file.

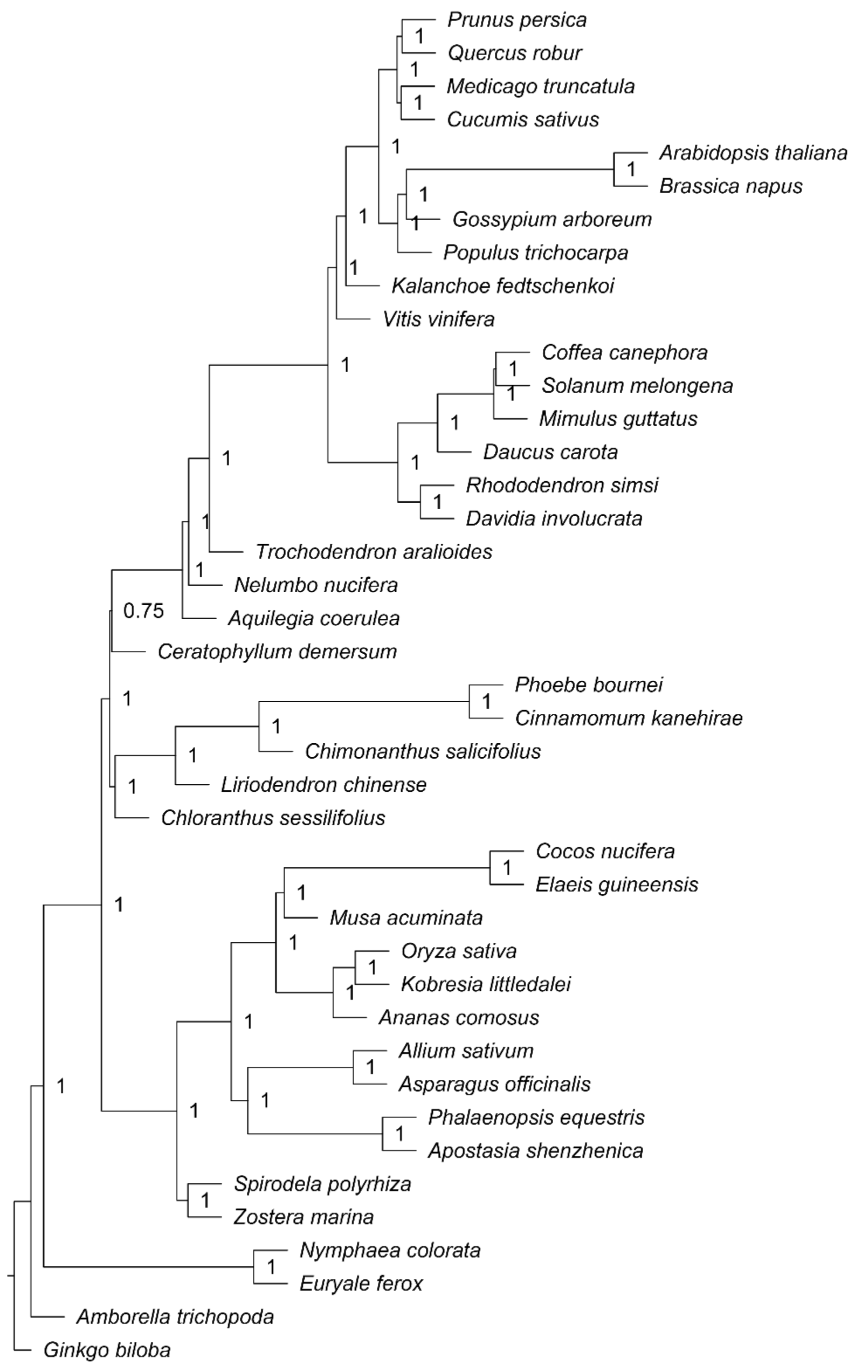

Supplementary Figure 21. ASTRAL tree of the 42 species with the 'mostly' single-copy orthologous.

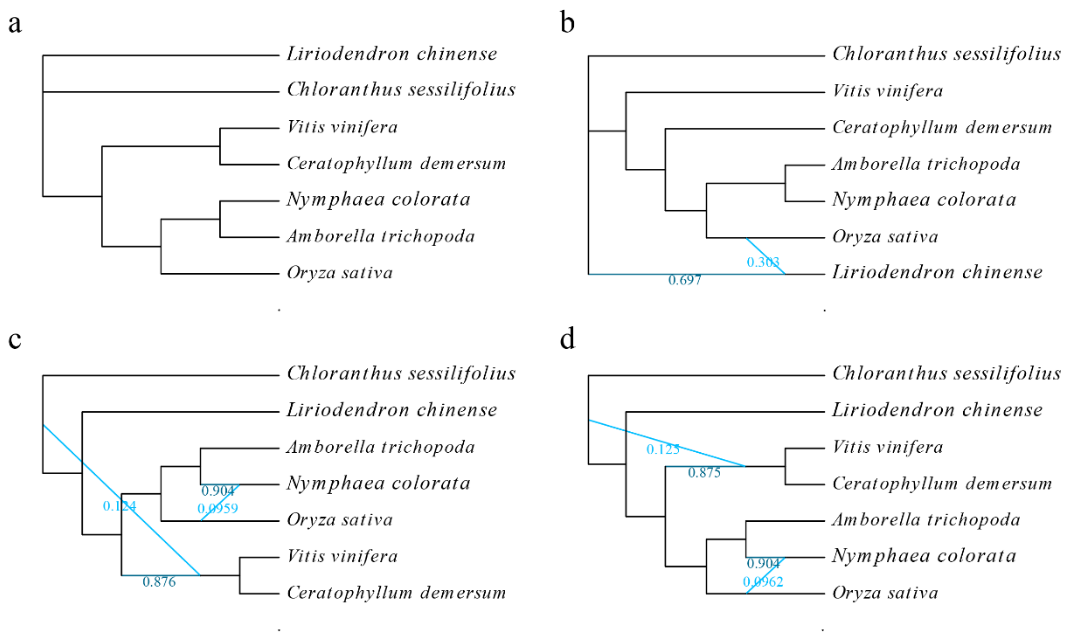

Supplementary Figure 22. The likely hybrid events among the seven species that inferred by PhyloNetworks. The blue lines represent the predicted hybrid events and the numbers on the blue line represent the proportion of the mixture. (a)-(d) Representing the number of hybridizations from 0-3. And for (d) even maximal hybridization set to 3, only 2 events were detected.

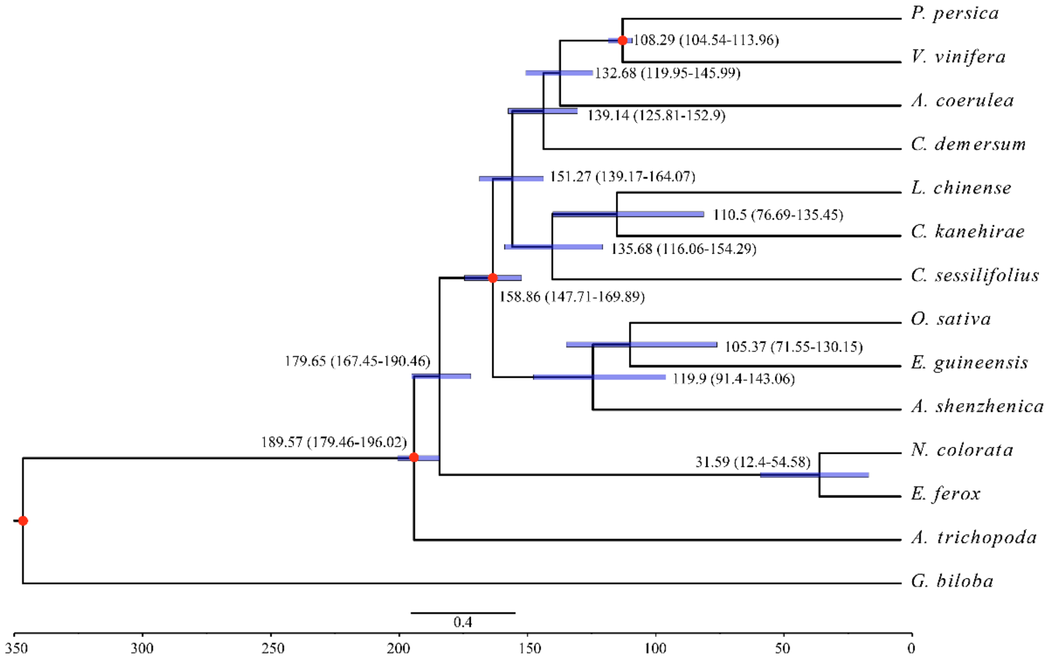

Supplementary Figure 23. Divergence time and gene family expansion and contraction estimated among 14 species. Divergence estimates (Mya, million years ago) are indicated above nodes and the blue nodal bars show 95% confidence intervals. The red dots correspond to calibration points as described in the methods.

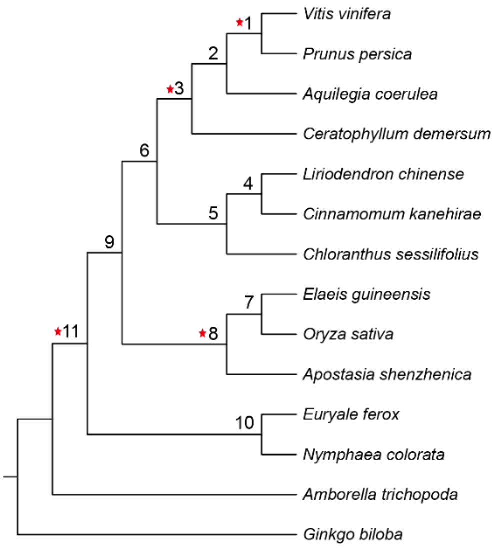

Internal branch ID	Frequence of minor topology 1		Frequence of minor topology 2		χ^2	*P* value
	Observed	Simulated	Observed	Simulated		
1	41	902	35	901	0.448	5.03E-01
2	247	1,806	59	1,787	104.910	1.28E-24
3	554	6,670	479	6,533	3.711	5.41E-02
4	208	2,251	123	2,079	14.534	1.38E-04
5	529	4,695	302	4,676	56.148	6.72E-14
6	540	5,910	386	5,801	21.161	4.22E-06
7	524	5,053	302	5,016	53.675	2.37E-13
8	22	374	21	353	0.001	9.71E-01
9	202	1,908	79	1,906	13.067	3.01E-04
10	0	5	0	3	-	-
11	430	4,907	391	4,846	1.289	2.56E-01

Supplementary Figure 24. Chi-squared test of the frequency of two minor topologies between the observed gene trees and simulated gene trees (Phybase with ILS). The two-sided chi-squared test was used there. The four internal branches with the red star represent no significant difference between the observed and simulated dataset, which indicates ILS is the major factor causing the discordance of these internal branches.

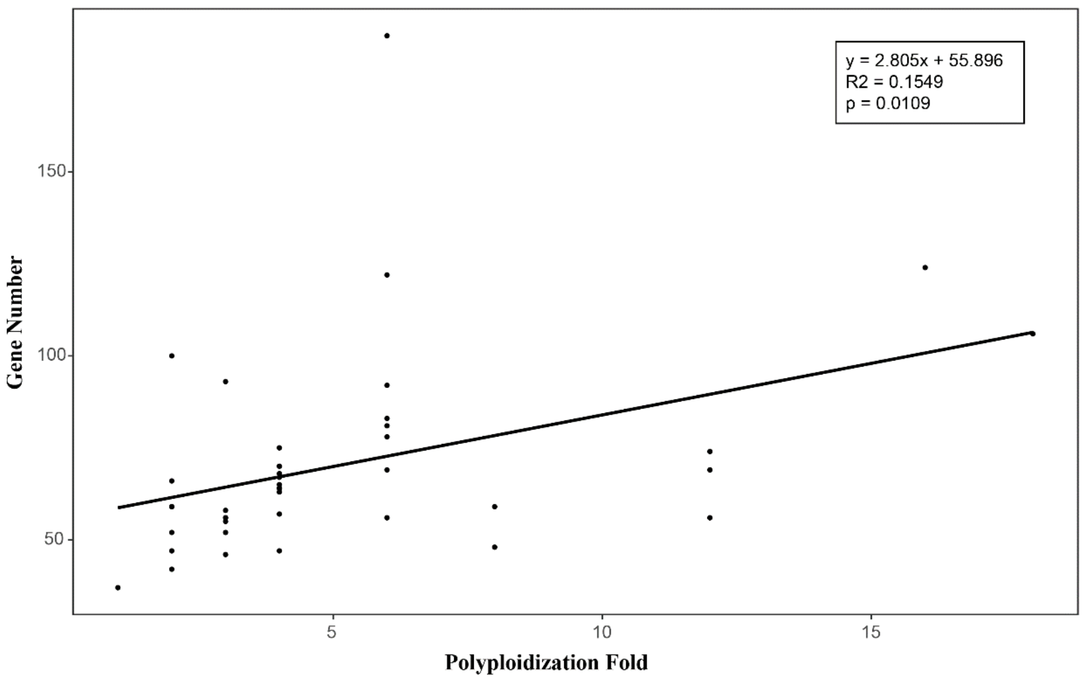

Supplementary Figure 25. The number of flowering-related genes is positively correlated with the WGD events across the angiosperms. The linear fitting was performed between flowering-related gene number and polyploidization fold by the function of ‘lm()’ in R. Source data are provided as a Source Data file.

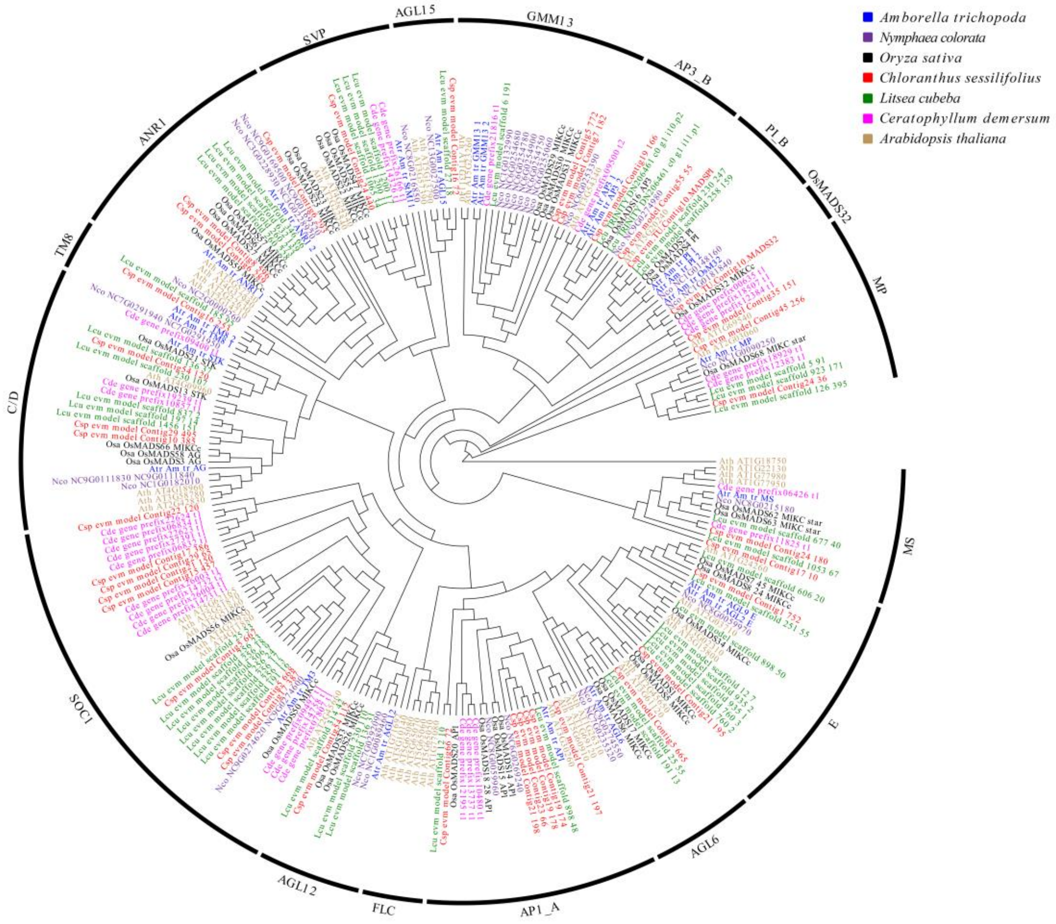

Supplementary Figure 26. Phylogenetic relationships of type II MADS-box genes in *Amborella trichopoda, Nymphaea colorata, Oryza sativa, Chloranthus sessilifolius, Litsea cubeba, Ceratophyllum demersum,* and *Arabidopsis thaliana*. Source data are provided as a Source Data file.

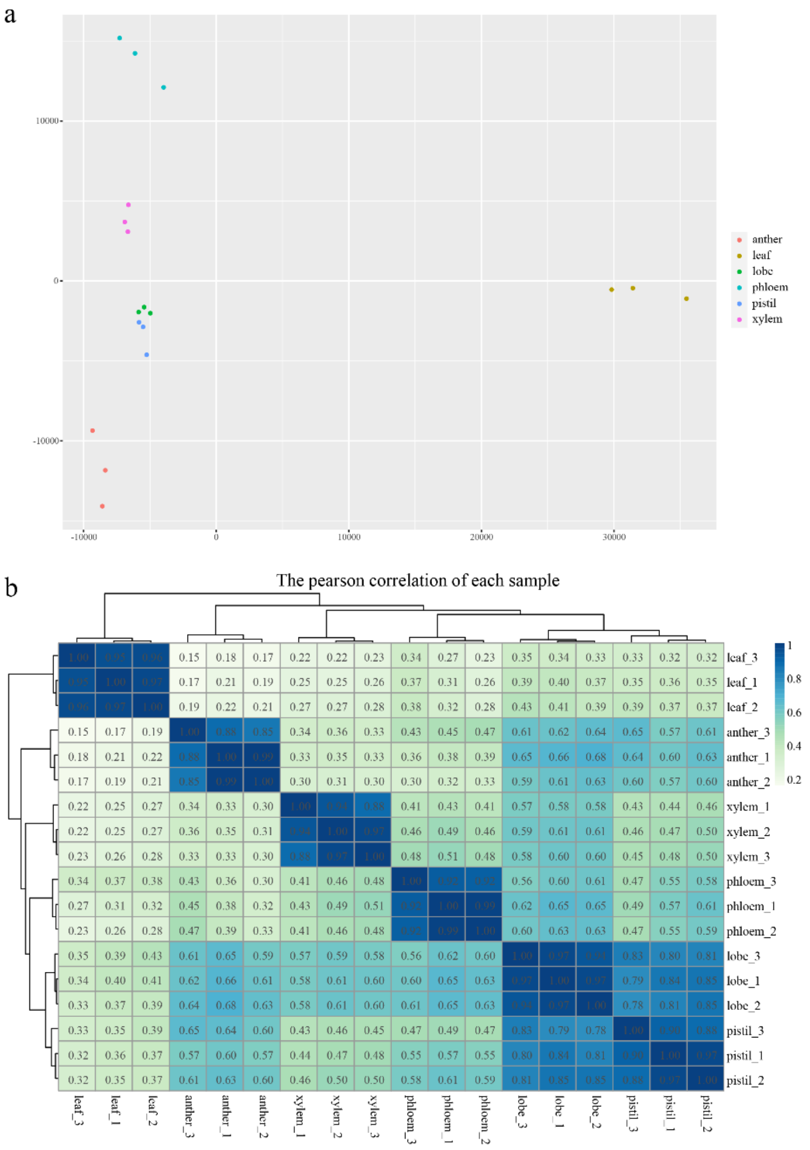

Supplementary Figure 27. Multidimensional scaling (MDS) plot (a), and the Pearson correlation analysis (b) of all the samples.

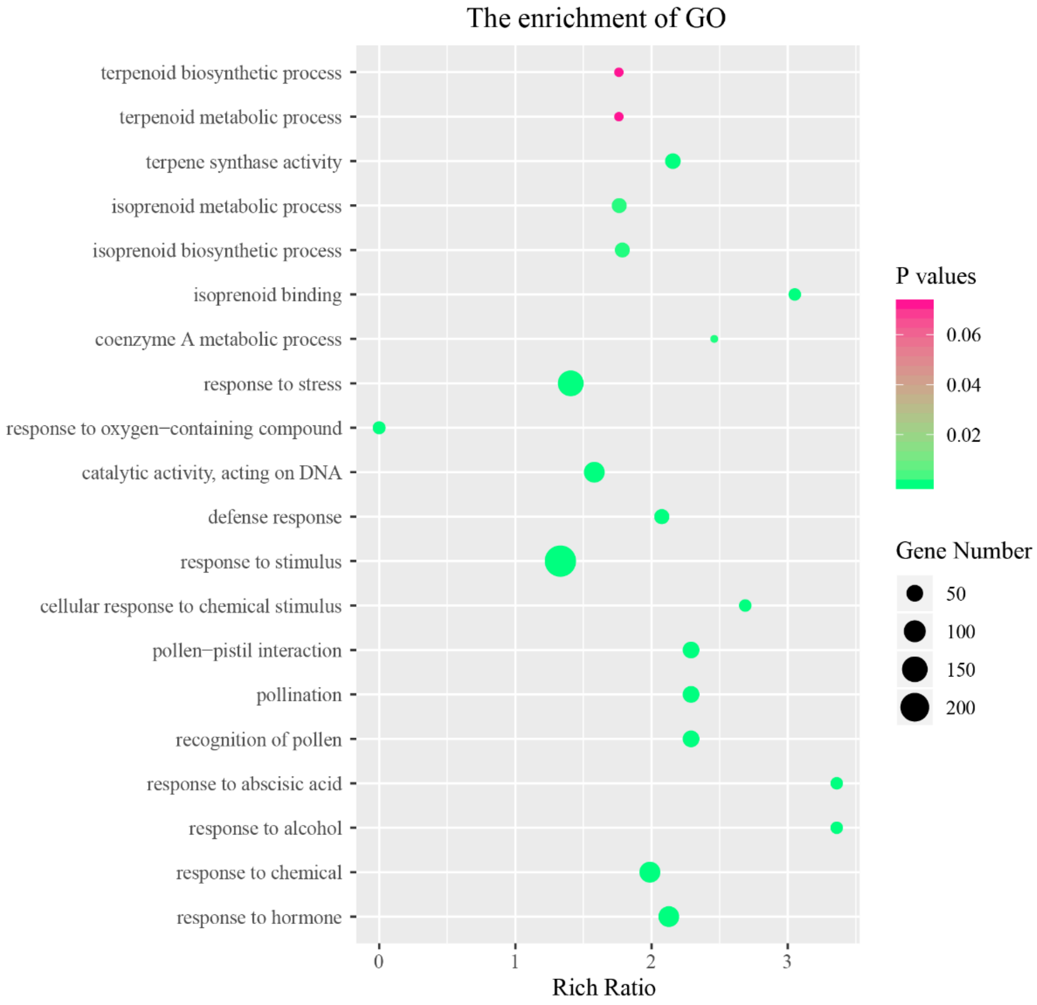

Supplementary Figure 28. The Gene Ontology (GO) enrichment analysis of expansion genes. The significantly enriched GO terms for genes with expansion.

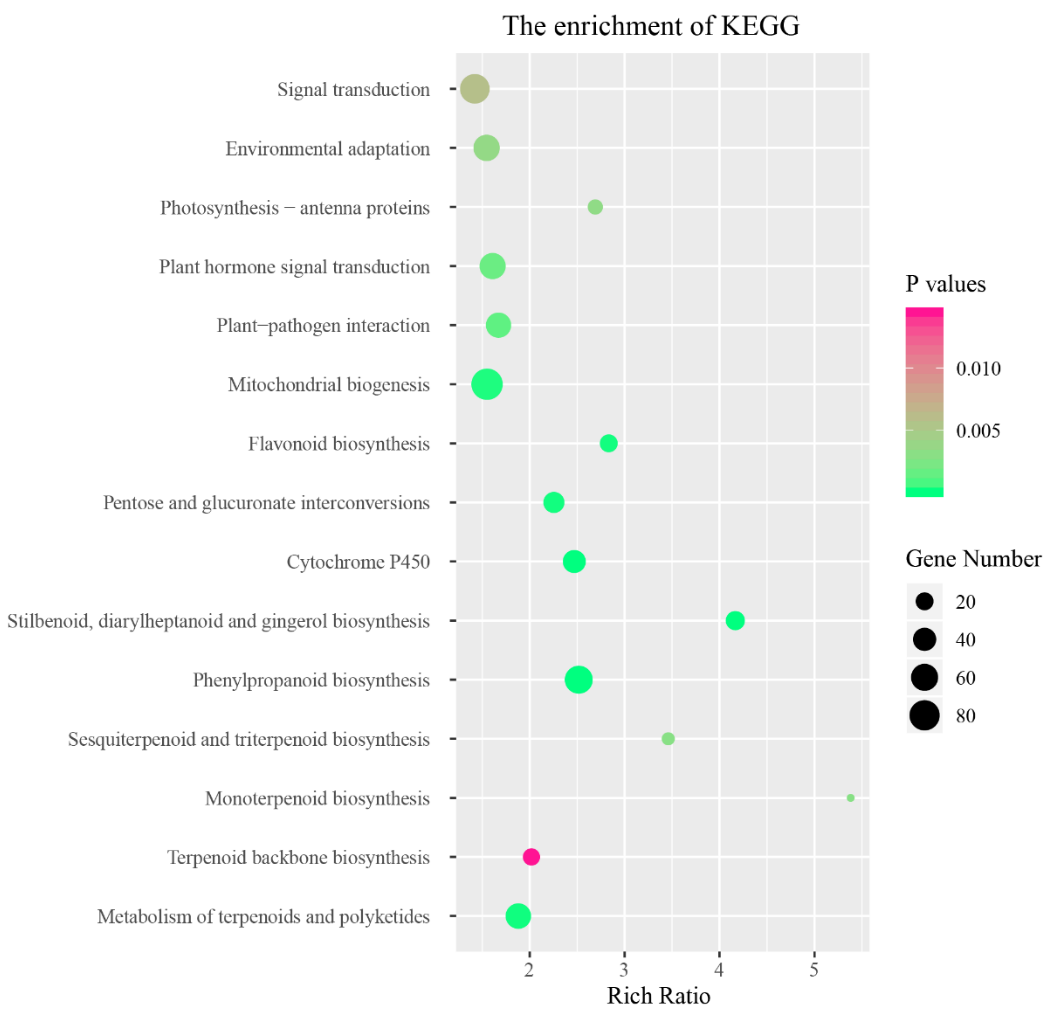

Supplementary Figure 29. The Kyoto Encyclopedia of Genes and Genomes (KEGG) enrichment analysis of expansion genes. The significantly enriched KEGG terms for genes with expansion.

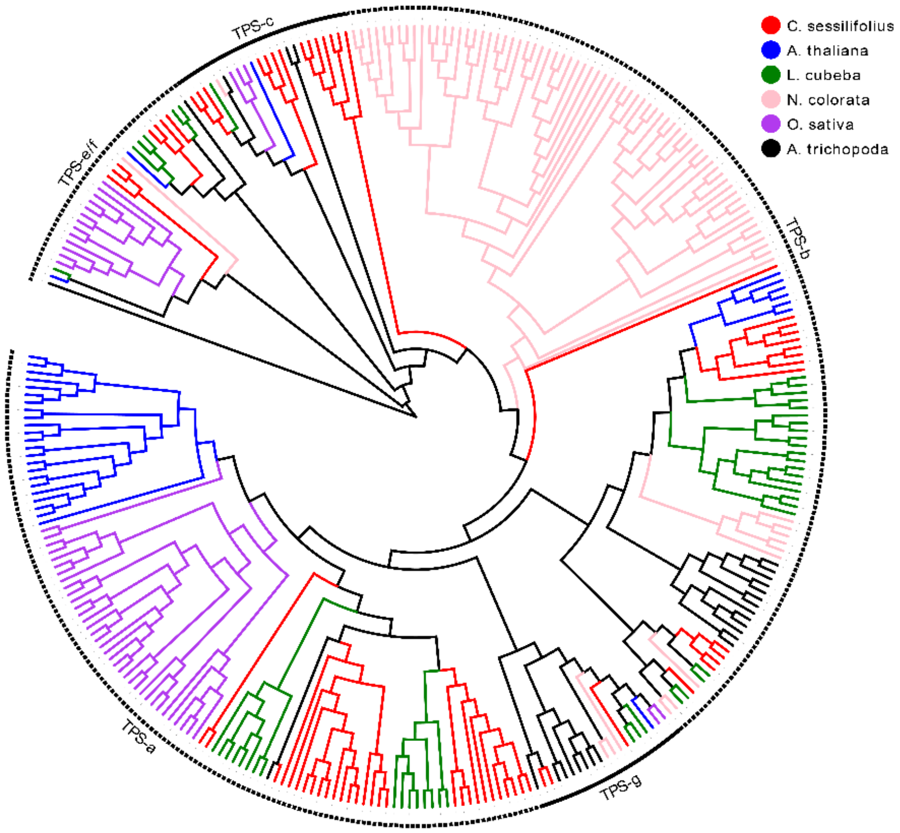

Supplementary Figure 30. Phylogeny placements of TPS genes in *Amborella trichopoda, Arabidopsis thaliana, Chloranthus sessilifolius, Litsea cubeba, Nymphaea colorata* and *Oryza sativa*. Source data are provided as a Source Data file.

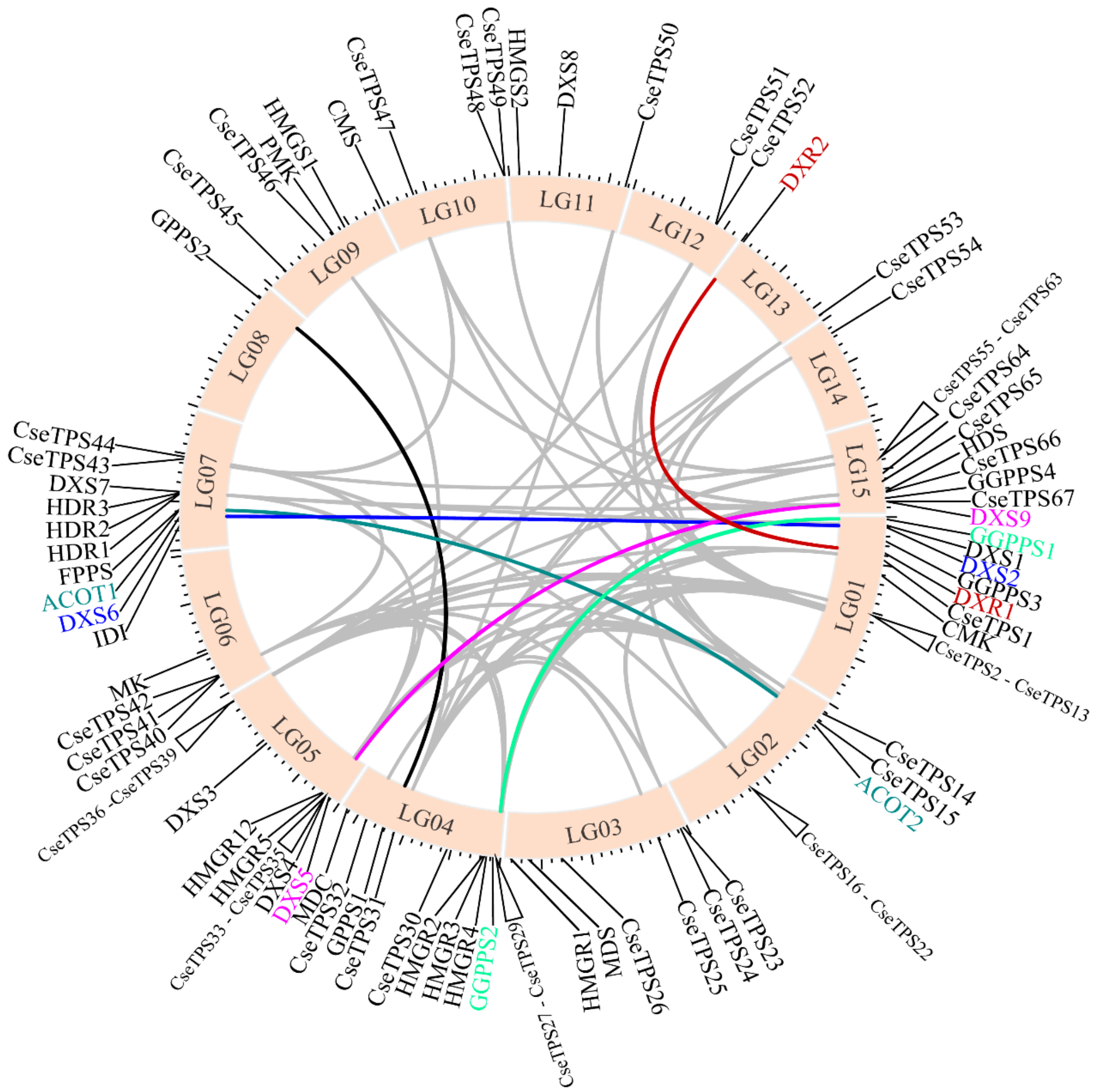

Supplementary Figure 31. Schematic representation of the synteny relationship and positions of key genes involved in terpenoid biosynthesis in *Chloranthus sessilifolius*. The genes in the MEP and MVA pathways are marked in black (*GPPS1* and *GPPS2*), red (*DXR1* and *DXR2*), green (*ACOT1* and *ACOT2*), pink (*DXS5* and *DXS9*), blue (*DXS2* and *DXS6*) and light green (*GGPPS1* and *GGPPS2*), those of which generated by WGD.

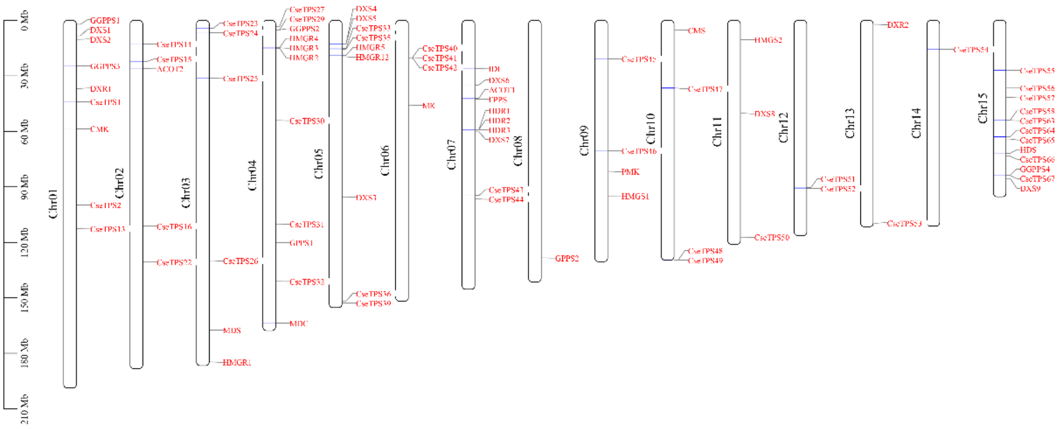

Supplementary Figure 32. Chromosome location of terpene biosynthesis genes in *Chloranthus sessilifolius*. Most genes were oriented by tandem duplication.

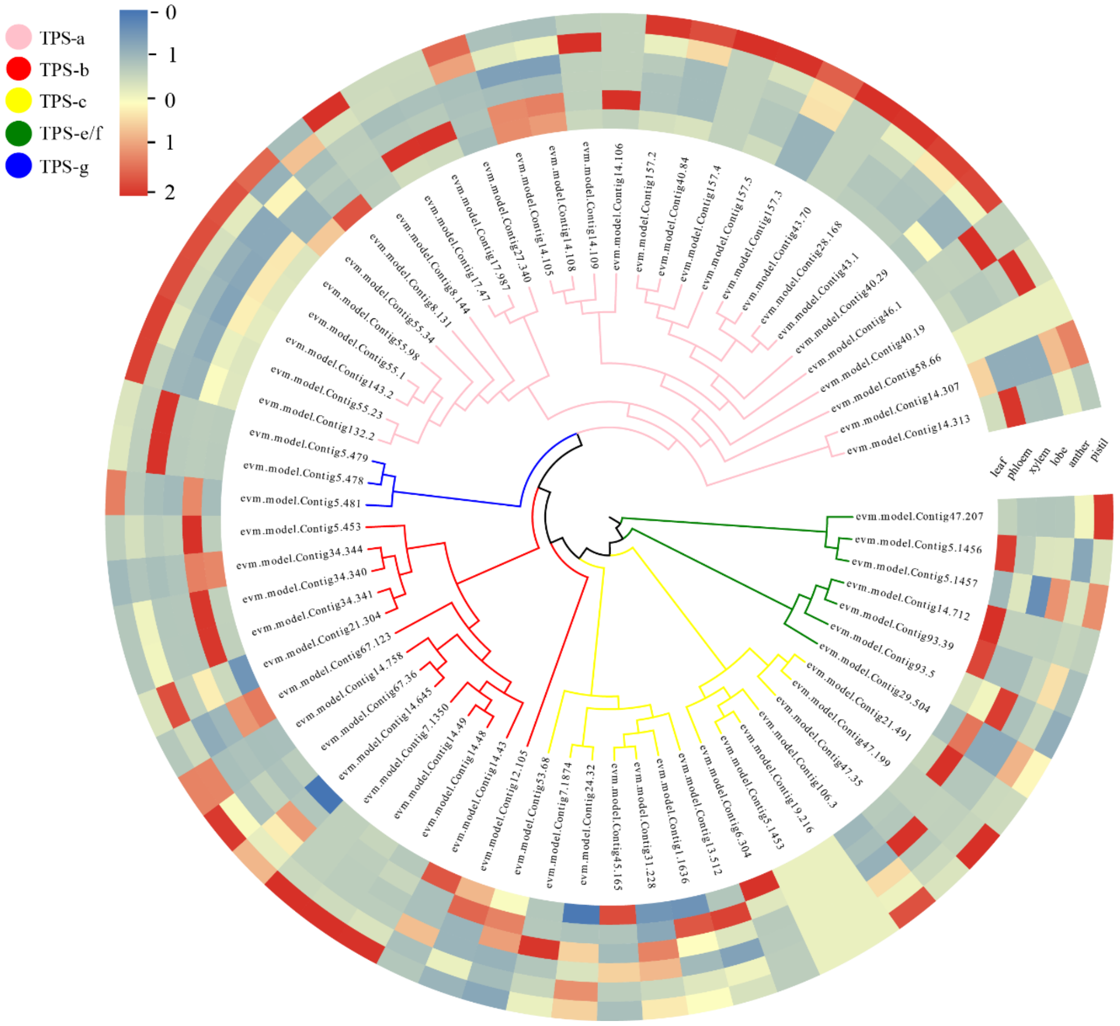

Supplementary Figure 33. Phylogenetic and expression analysis of TPS genes in *Chloranthus sessilifolius*. The expression levels were normalized by row. Source data are provided as a Source Data file.

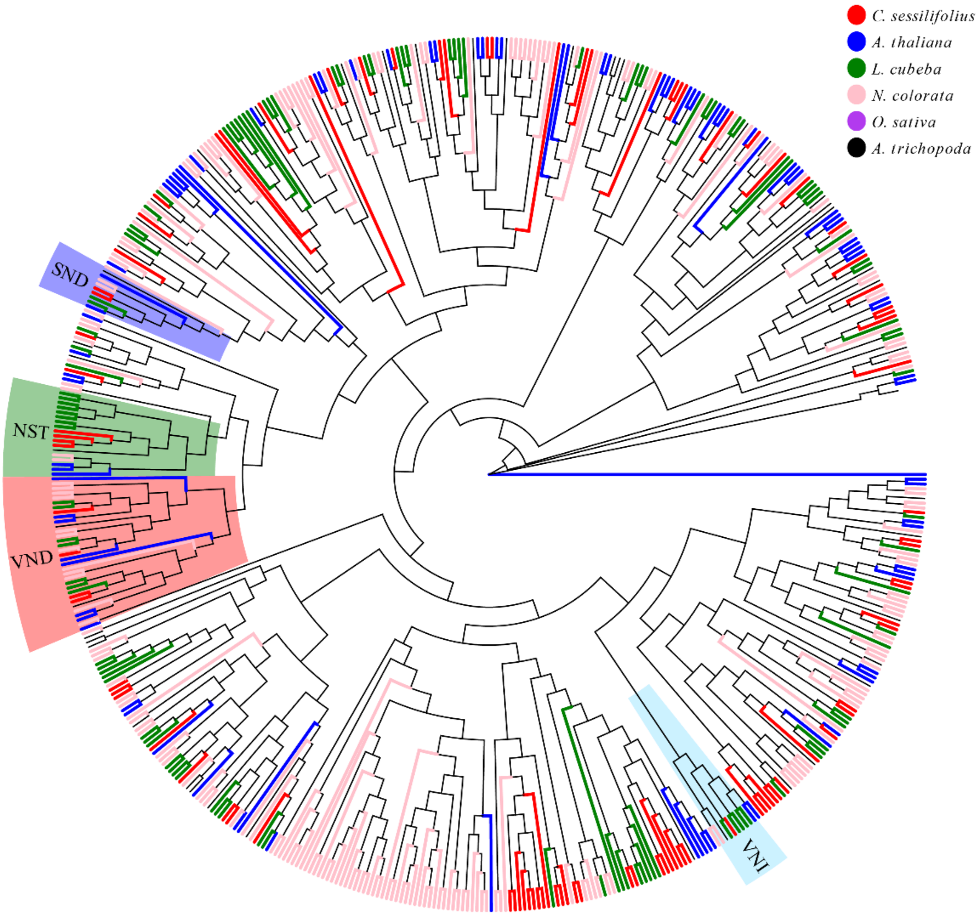

Supplementary Figure 34. Analysis of NAC transcription factors in six species. The colors of the terminal branches represent the different species. The different colors of shading represent the four sub-families. Source data are provided as a Source Data file.

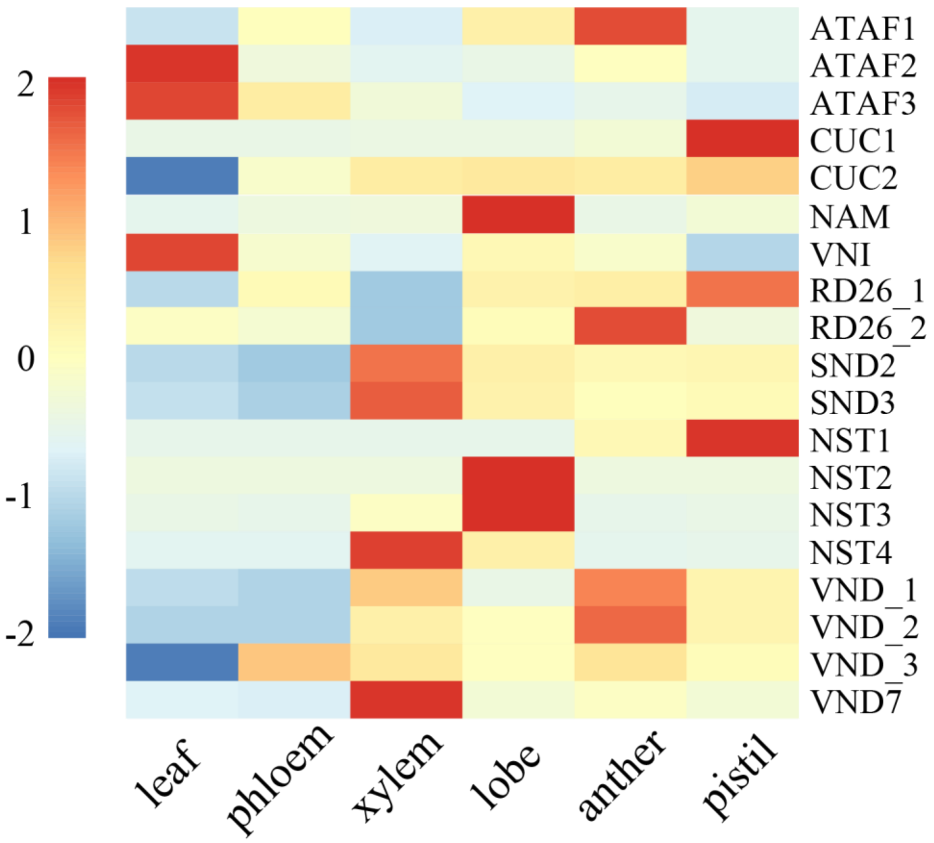

Supplementary Figure 35. Expression patterns of NAC genes in *Chloranthus sessilifolius*. The expression level was normalized by row. Source data are provided as a Source Data file.

Supplementary Table 1. The total clean sequencing data for *Chloranthus sessilifolius*.

Type	Platform	Library type/tissue	Reads number	Data size (Gb)	Read N50 (bp)
Illumina short reads	HiSeq X	Paired	47,326,782	100.00	-
ONT long reads	PromethION	Single	7,697,918	207.20	36,367
Hi-C reads	HiSeq X (raw)	Paired	1,722,139,556	249.51	-
transcriptome reads	MGI2000	Leaf_1	47,027,170	5.3	-
	MGI2000	Leaf_2	46,975,182	5.3	-
	MGI2000	Leaf_3	47,158,642	5.3	-
	MGI2000	Lobe_1	46,263,118	4.6	-
	MGI2000	Lobe_2	47,054,190	4.6	-
	MGI2000	Lobe_3	40,818,196	4.6	-
	MGI2000	Anther_1	40,557,374	5.1	-
	MGI2000	Anther_2	40,394,916	5.2	-
	MGI2000	Anther_3	40,682,810	4.6	-
	MGI2000	Pistil_1	46,920,244	5.2	-
	MGI2000	Pistil_2	46,412,750	5.1	-
	MGI2000	Pistil_3	40,894,254	4.6	-
	MGI2000	Xylem_1	49,063,240	5.5	-
	MGI2000	Xylem_2	46,424,916	5.1	-
	MGI2000	Xylem_3	47,757,020	5.3	-
	MGI2000	Phloem_1	46,121,700	4.7	-
	MGI2000	Phloem_2	46,859,868	5.2	-
	MGI2000	Phloem_3	46,495,462	5.2	-

Supplementary Table 2. Summary of *Chloranthus sessilifolius* contig leveled assembly.

	Size (MB)	Number
N90	10.60	55
N50	53.74	15
Longest	158.17	-
Shortest	0.04	-
Total Contigs	2,168.73	181
Short reads mapping rate	99.93%	

Supplementary Table 3. Summary of *Chloranthus sessilifolius* chromosome leveled assembly.

Chromosome	Length(bp)	Contig number
Chr01	199,165,573	9
Chr02	188,608,297	13
Chr03	186,975,679	10
Chr04	168,062,048	14
Chr05	155,683,883	7
Chr06	152,173,654	17
Chr07	145,775,264	11
Chr08	141,750,234	5
Chr09	130,815,930	16
Chr10	129,955,800	17
Chr11	121,397,826	9
Chr12	116,714,875	16
Chr13	111,892,785	16
Chr14	111,622,958	11
Chr15	95,708,319	16
Total	2,156,303,125	187

Supplementary Table 4. Evaluation of the *Chloranthus sessilifolius* genome completeness using data set of RNA transcripts.

Data set	Length type	Number	Total length	Covered by assembly (%)	With > 90% sequence in one scaffold		With > 50% sequence in one scaffold	
					Number	Percentage (%)	Number	Percentage (%)
**All**	>200bp	456,633	340,844,754	97.78	416,417	91.19	445,910	97.65
	>500bp	182,475	255,301,947	98.52	164,818	90.32	179,476	98.36
	>1,000bp	92,211	192,800,095	99.34	84,251	91.37	91,479	99.21
**Leaf**	>200bp	139,996	139,566,681	99.51	130,422	93.16	139,185	99.42
	>500bp	76,536	119,241,517	99.9	71,011	92.78	76,382	99.79
	>1,000bp	46,279	97,773,017	99..99	42,869	92.63	46,235	99.9
**Lobe**	>200bp	163,102	146,429,450	99.87	151,473	92.87	162,769	99.79
	>500bp	78,288	119,771,882	99.97	72,165	92.18	78,206	99.89
	>1,000bp	46,140	97,165,677	99.99	42,622	92.38	46,104	99.92
**Anther**	>200bp	193,912	161,015,016	96.55	174,404	89.94	186,922	96.4
	>500bp	86,768	127,218,046	97.8	78,423	90.38	84,680	97.59
	>1,000bp	47,654	99,925,331	99.15	43,913	92.15	47,167	98.98
**Pistil**	>200bp	193,912	161,015,016	97.21	174,404	90	186,922	96.88
	>500bp	86,768	127,218,046	98.32	78,423	90.39	84,680	98.45
	>1,000bp	47,654	99,925,331	99.68	43,913	92.55	47,167	99.12

Supplementary Table 5. Comparison of gene space of the *Chloranthus sessilifolius* genomes with other genomes.

Species	Total genes	Average CDS length	Average gene length	Average exon length	Average exons per gene	Average intron length
C. sessilifolius	34,065	1,195.18	19,506.37	202.34	5.91	3,731.74
A. trichopoda	26,834	945.62	5,660.53	231.34	4.08	1,527.11
G. biloba	41,309	1,177.60	25,619.24	279.88	4.2	7,618.97
C. kanehirae	27,859	1,311.88	7,602.28	242.59	5.4	1,427.15
N. colorata	31,363	1,136.50	4,968.82	278	5.53	755.63
O. sativa	42,148	1,113.15	2,841.77	327.6	4.43	405.09
C. demersum	25,323	1,134.24	11,900.30	220.28	5.7	2,262.78
V. vinifera	25,198	1,349.34	6,536.86	328.92	5.56	1,030.78

Supplementary Table 6. Functional annotation of the predicted genes for *Chloranthus sessilifolius*.

	**Database**	**Number**	**Percent (%)**
Annotated	InterPro	27,146	84.65
	GO	15,418	48.08
	NR	26,121	81.46
	Swissprot	20,210	63.02
	KEGG	7,009	21.86
Total	-	31,312	97.65

Supplementary Table 7. Summary of non-protein-coding gene annotations in the *Chloranthus sessilifolius* genome assembly.

Software	Type	Number	Average_length
tRNAscan-SE	tRNA	889	100
BLAST	rRNA	767	242.65
Infernal	miRNA	296	111.21
	snRNA	7,827	106.74

Supplementary Table 8. Prediction of transposable elements in the assembled *Chloranthus sessilifolius* genomes.

	Length (bp)	Percentage of genome (%)
SINE	151,853	0.007
LINE	80,074,142	3.69
LTR	1,188,640,928	54.81
DNA	139,413,831	6.43
Tandem repeats	23,527,611	1.08
Unknown	329,329,218	15.19
Total	1,761,137,583	81.04

Supplementary Table 9. The results of a chi-squared test of the TE accounted for the proportion of genic region and intergenic. The chi-squared tests were two side tests.

		Length of genic region	Length of intergenic
Content of non-TE		374,041,279	334,100,958
Content of TE		389,153,506	1,071,432,893
Chi-squared test	*x2*	143,283,790	
	P	2.20E-16	

Supplementary Table 10. The results of a chi-squared test of the TE accounted for the proportion of exonic region and intronic region. The chi-squared tests were two side tests.

		Length of the exonic region	Length of the intronic region
Content of non-TE		36,862,756	262,805,547
Content of TE		3,591,703	338,754,868
Chi-squared test	*x2*	34,266,444	
	P	2.20E-16	

Supplementary Table 11. The genomic information of used species in phylogeny analyses.

Species name	Number of genes	Source
Ginkgo biloba	31,960	10.1186/s13742-016-0154-1
Amborella trichopoda	26,844	10.1126/science.1241089
Nymphaea colorata	31,363	10.1038/s41586-019-1852-5
Euryale ferox	40,197	10.1038/s41477-020-0594-6
Ananas comosus	26,508	10.1038/ng.3435
Kobresia littledalei	22,441	10.1038/s41597-020-0518-3.
Musa acuminata	34,997	10.1038/nature11241
Apostasia shenzhenica	21,739	10.1038/nature23897
Asparagus officinalis	25,223	10.1038/s41467-017-01064-8
Allium sativum	57,561	10.1016/j.molp.2020.07.019
Spirodela polyrhiza	19,414	10.1073/pnas.1910401116
Zostera marina	20,284	10.1038/nature16548
Cocos nucifera	28,003	10.1089/omi.2020.0147
Elaeis guineensis	24,382	10.1038/nature12309
Oryza sativa	42,156	10.1186/1939-8433-6-4
Chloranthus sessilifolius	34,065	this study
Piper nigrum	62,621	10.1038/s41467-019-12607-6
Chimonanthus salicifolius	36,291	10.1186/s13059-020-02088-y
Phoebe bournei	31,743	10.1038/s41438-020-00368-z
Cinnamomum kanehirae	26,513	10.1038/s41477-018-0337-0
Liriodendron chinense	35,264	10.1038/s41477-018-0323-6
Ceratophyllum demersum	29,717	10.1038/s41477-020-0594-6
Aquilegia coerulea	29,880	10.7554/eLife.36426
Vitis vinifera	25,222	10.1038/nature06148
Prunus persica	28,892	10.1038/ng.2586
Nelumbo nucifera	23,784	10.1111/tpj.12313
Trochodendron aralioides	34,714	10.1186/s13059-020-02198-7
Coffea canephora	25,430	10.1126/science.1255274
Rhododendron simsi	32,999	10.1038/s41467-020-18771-4
Davidia involucrata	42,433	10.1111/1755-0998.13138
Mimulus guttatus	28,118	10.1073/pnas.1319032110
Daucus carota	31,785	10.1038/ng.3565
Solanum melongena	35,615	10.1111/tpj.15313
Kalanchoe fedtschenkoi	30,818	10.1038/s41467-017-01491-7
Cucumis sativus	19,009	10.1038/ng.475
Populus trichocarpa	34,586	10.1126/science.1128691
Medicago truncatula	50,731	10.1038/nature10625
Quercus robur	25,591	10.1038/s41477-018-0172-3
Brassica napus	82,660	10.1126/science.1253435
Arabidopsis thaliana	27,307	10.1038/s41467-018-03016-2
Gossypium arboreum	33,173	10.1038/ng.2987

Supplementary Table 12. MADS-Box genes in *Chloranthus sessilifolius.*

Gene ID	Name	Protein	Type	Putative subfamily
evm.model.Contig19.174	AP1_1	268	MIKCc	A
evm.model.Contig19.178	AP1_2	869	MIKCc	A
evm.model.Contig21.197	AP1_3	63	MIKCc	A
evm.model.Contig21.198	AP1_4	174	MIKCc	A
evm.model.Contig23.66	AP1_5	460	MIKCc	A
evm.model.Contig66.72	AP1_6	99	MIKCc	A
evm.model.Contig19.166	AP3_1	220	MIKCc	B
evm.model.Contig19.165	AP3_2	102	MIKCc	B
evm.model.Contig10.MADSPI	PI	210	MIKCc	B
evm.model.Contig29.495	AG_1	170	MIKCc	C/D
evm.model.Contig10.385	AG_2	289	MIKCc	C/D
evm.model.Contig54.120	STK	223	MIKCc	C/D
evm.model.Contig1.752	SEP3	215	MIKCc	E
evm.model.Contig21.195	SEP1	241	MIKCc	E
evm.model.Contig6.289	ANR1_1	78	MIKCc	ANR1
evm.model.Contig6.293	ANR1_2	167	MIKCc	ANR1
evm.model.Contig8.596	ANR1_3	241	MIKCc	ANR1
evm.model.Contig17.386	SOC1 _1	62	MIKCc	SOC1/TM3
evm.model.Contig17.387	SOC1 _2	158	MIKCc	SOC1/TM3
evm.model.Contig21.150	SOC1 _3	95	MIKCc	SOC1/TM3
evm.model.Contig29.65	SOC1 _4	258	MIKCc	SOC1/TM3
evm.model.Contig5.667	SOC1 _5	80	MIKCc	SOC1/TM3
evm.model.Contig5.668	SOC1 _6	202	MIKCc	SOC1/TM3
evm.model.Contig16.253	TM8	248	MIKCc	TM8
evm.model.Contig54.112	AGL12	174	MIKCc	AGL12
evm.model.Contig5.665	AGL6	244	MIKCc	AGL6
evm.model.Contig7.182	AGL32_1	236	MIKCc	GMM13/Bs/AGL3
evm.model.Contig5.772	AGL32_2	175	MIKCc	GMM13/Bs/AGL3
evm.model.Contig16.72	AGL15	241	MIKCc	AGL15
evm.model.Contig24.440	SVP	558	MIKCc	SVP
evm.model.Contig10.MADS32	OsMADS32	198	MIKCc	OsMADS32
evm.model.Contig17.10	S1	301	MIKC*	S
evm.model.Contig24.180	S2	310	MIKC*	S
evm.model.Contig24.36	P1	467	MIKC*	P
evm.model.Contig35.151	P2	294	MIKC*	P
evm.model.Contig45.256	P3	572	MIKC*	P
evm.model.Contig6.377	Mα_1	750	Type I	Mα

evm.model.Contig37.55	Mα_2	206	Type I	Mα
evm.model.Contig54.266	Mα_3	167	Type I	Mα
evm.model.Contig28.322	Mα_4	177	Type I	Mα
evm.model.Contig28.288	Mα_5	208	Type I	Mα
evm.model.Contig28.321	Mα_6	196	Type I	Mα
evm.model.Contig28.323	Mα_7	222	Type I	Mα
evm.model.Contig6.1345	Mα_8	88	Type I	Mα
evm.model.Contig28.287	Mα_9	215	Type I	Mα
evm.model.Contig17.136	Mα_10	164	Type I	Mα
evm.model.Contig6.376	Mα_11	214	Type I	Mα
evm.model.Contig6.375	Mα_12	215	Type I	Mα
evm.model.Contig15.457	Mb_1	579	Type I	Mb
evm.model.Contig2.327	Mb_2	180	Type I	Mb
evm.model.Contig22.120	Mb_3	473	Type I	Mb
evm.model.Contig6.1662	Mb_4	530	Type I	Mb
evm.model.Contig1.472	Mb_5	273	Type I	Mb
evm.model.Contig1.504	Mb_6	272	Type I	Mb
evm.model.Contig66.45	Mb_7	282	Type I	Mb
evm.model.Contig28.180	Mγ_1	217	Type I	Mγ
evm.model.Contig28.181	Mγ_2	219	Type I	Mγ
evm.model.Contig80.60	Mγ_3	231	Type I	Mγ

Supplementary Table 13. The expression (FPKM) of MADS-Box genes in *Chloranthus sessilifolius.*

geneID	xylem	phloem	leaf	lobe	anther	pistil
AP1_1	0.82	1.11	0.38	0.83	0.39	0.92
AP1_2	0.36	0.55	0.35	1.17	0.59	1.57
AP1_3	8.01	13.60	1.61	267.81	19.20	33.91
AP1_4	10.67	21.50	1.70	362.48	22.26	48.22
AP1_5	0.11	0.18	0.00	0.45	0.00	0.18
AP1_6	1.15	0.22	1.73	16.15	6.27	3.65
AP3_1	2.19	2.30	0.34	25.85	1188.66	49.29
AP3_2	0.00	0.00	0.00	0.07	1.45	0.00
PI	16.79	13.03	19.02	101.02	312.28	59.33
AG_1	0.00	0.00	3.87	2.11	2.11	114.96
AG_2	10.01	20.15	9.95	66.70	281.65	100.40
STK	0.13	0.11	0.14	15.38	27.10	249.58
SEP3	0.14	0.72	0.00	149.85	423.99	506.22
SEP1	44.76	35.46	38.52	159.82	117.28	96.47
ANR1_1	0.00	1.28	2.91	0.00	0.14	0.00
ANR1_2	0.10	1.35	1.08	0.08	0.00	0.08
ANR1_3	0.05	0.00	0.00	0.00	0.05	0.00
SOC1_1	42.98	145.71	162.40	11.71	67.14	4.60
SOC1_2	42.09	139.59	148.49	8.57	51.50	3.60
SOC1_3	8.08	9.22	1.16	8.54	2.96	9.06
SOC1_4	0.09	0.02	0.00	0.07	0.00	0.05
SOC1_5	79.25	127.70	171.53	0.06	3.75	8.39
SOC1_6	99.06	164.07	213.81	0.15	5.96	10.29
TM8	2.58	1.74	0.62	18.79	1.03	4.99
AGL12	5.40	0.00	0.10	1.54	0.14	0.00
AGL6	5.30	5.54	4.21	172.95	8.18	473.52
AGL32_1	0.00	0.00	0.00	0.09	0.05	44.57
AGL32_2	0.00	0.00	0.00	0.15	0.05	44.99
AGL15	7.80	7.09	7.41	4.58	4.65	9.18
SVP	53.47	42.27	56.81	0.18	0.00	0.00
OsMADS32	0.05	0.00	0.00	0.38	0.02	0.19
S1	0.02	0.00	0.00	0.10	0.06	0.07
S2	0.05	0.24	0.00	0.21	2.83	0.57
P1	2.59	3.68	3.26	3.90	2.79	3.10
P2	0.01	0.01	0.03	0.03	0.27	0.13
P3	4.05	7.57	3.93	4.53	4.62	5.07

Supplementary Table 14. Gene ontology (GO) enrichment analysis of the expanded gene families in *Chloranthus sessilifolius*. The one-sided Fisher's Exact Test was used (by setting alternative = 'greater') and then all the *p* values were performed the multiple corrections under the 'Benjamini & Hochberg' method.

GO ID	Type	GO Terms	# of enriched genes	# of genes in background	Adjusted *p*-value
GO:0000723	BP	telomere maintenance	66	88	1.04E-13
GO:0060249	BP	anatomical structure homeostasis	66	88	1.04E-13
GO:0032200	BP	telomere organization	66	88	1.04E-13
GO:0009719	BP	response to endogenous stimulus	87	148	3.12E-13
GO:0009725	BP	response to hormone	87	148	3.12E-13
GO:0010033	BP	response to organic substance	87	149	3.47E-13
GO:0042221	BP	response to chemical	89	162	1.92E-11
GO:0097305	BP	response to alcohol	26	28	6.51E-11
GO:0097306	BP	cellular response to alcohol	26	28	6.51E-11
GO:0009737	BP	response to abscisic acid	26	28	6.51E-11
GO:0009738	BP	abscisic acid-activated signaling pathway	26	28	6.51E-11
GO:0071215	BP	cellular response to abscisic acid stimulus	26	28	6.51E-11
GO:0043178	MF	alcohol binding	26	28	1.95E-10
GO:0010427	MF	abscisic acid binding	26	28	1.95E-10
GO:0019840	MF	isoprenoid binding	26	28	1.95E-10
GO:0033293	MF	monocarboxylic acid binding	26	28	1.95E-10
GO:0042562	MF	hormone binding	26	28	1.95E-10
GO:0004864	MF	protein phosphatase inhibitor activity	27	31	1.95E-09
GO:0019212	MF	phosphatase inhibitor activity	27	31	1.95E-09
GO:0048544	BP	recognition of pollen	50	79	2.40E-09
GO:0009856	BP	pollination	50	79	2.40E-09
GO:0009875	BP	pollen-pistil interaction	50	79	2.40E-09
GO:0032870	BP	cellular response to hormone stimulus	26	32	1.84E-08
GO:0071310	BP	cellular response to organic substance	26	32	1.84E-08
GO:0071495	BP	cellular response to endogenous stimulus	26	32	1.84E-08
GO:0009733	BP	response to auxin	51	88	7.46E-08
GO:0010333	MF	terpene synthase activity	42	64	1.29E-07
GO:1901701	BP	cellular response to oxygen-containing compound	26	34	1.54E-07
GO:0070887	BP	cellular response to chemical stimulus	26	35	4.09E-07
GO:0003824	MF	catalytic activity	2313	7123	8.60E-07
GO:0050896	BP	response to stimulus	249	677	1.27E-06
GO:0006952	BP	defense response	39	68	6.81E-06
GO:0140097	MF	catalytic activity, acting on DNA	87	181	6.97E-06
GO:1901700	BP	response to oxygen-containing compound	28	43	8.87E-06

GO:0006950	BP	response to stress	152	391	1.46E-05
GO:0015936	BP	coenzyme A metabolic process	17	25	6.91E-04
GO:0008299	BP	isoprenoid biosynthetic process	38	77	8.81E-04
GO:0006720	BP	isoprenoid metabolic process	38	78	0.001235405
GO:0006721	BP	terpenoid metabolic process	18	37	0.072169829
GO:0016114	BP	terpenoid biosynthetic process	18	37	0.072169829

[1]MF: molecular function; BP: biological process; CC: cellular component.

Supplementary Table 15. KEGG enrichment analysis of the expanded gene families in *Chloranthus sessilifolius*. The one-sided Fisher's Exact Test was used (by setting alternative = 'greater') and then all the *p* values were performed the multiple corrections under the 'Benjamini & Hochberg' method.

Map ID	Map title	# of enriched genes	# of genes in background	Adjusted *p*-value
Map09109	Metabolism of terpenoids and polyketides	51	146	6.04E-05
Map00940	Phenylpropanoid biosynthesis	65	139	3.19E-12
Map00945	Stilbenoid, diarylheptanoid and gingerol biosynthesis	24	31	1.01E-10
Map00199	Cytochrome P450	39	85	2.94E-07
Map00040	Pentose and glucuronate interconversions	31	74	6.92E-05
Map00941	Flavonoid biosynthesis	20	38	7.02E-05
Map03029	Mitochondrial biogenesis	87	302	1.46E-04
Map04626	Plant-pathogen interaction	50	161	0.001320223
Map04075	Plant hormone signal transduction	55	184	0.001718984
Map00902	Monoterpenoid biosynthesis	5	5	0.002893592
Map00909	Sesquiterpenoid and triterpenoid biosynthesis	9	14	0.002958518
Map00196	Photosynthesis-antenna proteins	13	26	0.003388855
Map09159	Environmental adaptation	56	195	0.003573091
Map09132	Signal transduction	75	284	0.005811725
Map00900	Terpenoid backbone biosynthesis	18	48	0.014488175

Supplementary Table 16. The number of terpene synthesis-related enzymes in six species.

	Amborella trichopoda	*Nymphaea colorata*	*Oryza sativa*	*Chloranthus sessilifolius*	*Litsea cubeba*	*Arabidopsis thaliana*
DXS	3	1	3	9	7	3
DXR	1	1	1	2	1	1
MCT	1	1	1	1	1	1
CMK	1	1	1	1	1	1
MDS	1	1	1	1	1	1
HDS	1	1	1	1	2	1
HDR	1	2	2	3	2	1
AACT	1	2	2	2	5	2
HMGS	1	2	3	2	3	1
HMGR	1	2	3	12	4	2
MK	1	1	1	1	1	1
PMK	1	1	1	1	1	1
MPDC	1	1	2	1	1	2
IPPI	1	1	2	1	2	2
FPPS	1	1	5	1	4	2
GPPS	1	2	2	2	4	2
GGPPS	2	3	3	4	5	11
TPS-a	0	0	35	29	16	23
TPS-b	13	85	0	14	23	6
TPS-c	3	1	3	14	1	1
TPS-e/f	2	1	13	7	6	2
TPS-g	8	9	2	3	3	1

Supplementary Table 17. Enzymes involved in terpenoid biosynthesis pathways in *Chloranthus sessilifolius*.

	Gene ID	Abbreviation	Full name
MEP pathway	evm.model.Contig1.1746	DXS1	1-Deoxy-D-xylulose 5-phosphate synthase
	evm.model.Contig2.131	DXS2	1-Deoxy-D-xylulose 5-phosphate synthase
	evm.model.Contig34.375	DXS3	1-Deoxy-D-xylulose 5-phosphate synthase
	evm.model.Contig34.374	DXS4	1-Deoxy-D-xylulose 5-phosphate synthase
	evm.model.Contig46.155	DXS5	1-Deoxy-D-xylulose 5-phosphate synthase
	evm.model.Contig48.11	DXS6	1-Deoxy-D-xylulose 5-phosphate synthase
	evm.model.Contig6.1171	DXS7	1-Deoxy-D-xylulose 5-phosphate synthase
	evm.model.Contig6.1239	DXS8	1-Deoxy-D-xylulose 5-phosphate synthase
	evm.model.Contig8.578	DXS9	1-Deoxy-D-xylulose 5-phosphate synthase
	evm.model.Contig6.84	DXR1	1-Deoxy-D-xylulose 5-phosphate reductoisomerase
	evm.model.Contig15.679	DXR2	1-Deoxy-D-xylulose 5-phosphate reductoisomerase
	evm.model.Contig7.961	MCT	2-*C*-methyl-D-erythritol 4-phosphate cytidylyltransferase
	evm.model.Contig6.755	CMK	4-(Cytidine 5-diphospho)-2-*C*-methyl-D-erythritol kinase
	evm.model.Contig13.1039	MDS	2-*C*-methyl-D-erythritol 2,4-cyclodiphosphate synthase
	evm.model.Contig53.51	HDS	4-Hydroxy-3-methylbut-2-enyl-diphosphate synthase
	evm.model.Contig8.614	HDR1	4-Hydroxy-3-methylbut-2-enyl diphosphate reductase
	evm.model.Contig8.613	HDR2	4-Hydroxy-3-methylbut-2-enyl diphosphate reductase
	evm.model.Contig8.604	HDR3	4-Hydroxy-3-methylbut-2-enyl diphosphate reductase
MVA pathway	evm.model.Contig1.2441	AACT1	Acetyl-CoA *C*-acetyltransferase
	evm.model.Contig21.563	AACT2	Acetyl-CoA *C*-acetyltransferase
	evm.model.Contig17.784	HMGS1	3-Hydroxy-3-methylglutaryl-CoA synthase
	evm.model.Contig18.270	HMGS2	3-Hydroxy-3-methylglutaryl-CoA synthase
	evm.model.Contig13.1229	HMGR1	3-Hydroxy-3-methylglutaryl-CoA reductase
	evm.model.Contig68.52	HMGR2	3-Hydroxy-3-methylglutaryl-CoA reductase
	evm.model.Contig56.45	HMGR3	3-Hydroxy-3-methylglutaryl-CoA reductase
	evm.model.Contig56.41	HMGR4	3-Hydroxy-3-methylglutaryl-CoA reductase
	evm.model.Contig68.53	HMGR5	3-Hydroxy-3-methylglutaryl-CoA reductase
	evm.model.Contig47.170	HMGR6	3-Hydroxy-3-methylglutaryl-CoA reductase
	evm.model.Contig56.44	HMGR7	3-Hydroxy-3-methylglutaryl-CoA reductase
	evm.model.Contig56.37	HMGR8	3-Hydroxy-3-methylglutaryl-CoA reductase
	evm.model.Contig56.48	HMGR9	3-Hydroxy-3-methylglutaryl-CoA reductase
	evm.model.Contig56.32	HMGR10	3-Hydroxy-3-methylglutaryl-CoA reductase
	evm.model.Contig56.39	HMGR11	3-Hydroxy-3-methylglutaryl-CoA reductase
	evm.model.Contig34.1	HMGR12	3-Hydroxy-3-methylglutaryl-CoA reductase
	evm.model.Contig5.940	MK	MVA kinase
	evm.model.Contig17.73	PMK	Phospho-MVA kinase

	evm.model.Contig7.576	MPDC	Diphospho-MVA decarboxylase
Branch points	evm.model.Contig1.2615	IPPI	Isopentenyl diphosphate -isomerase
	evm.model.Contig1.1001	FPPS	Farnesyl diphosphate synthase
	evm.model.Contig7.1257	GPPS1	Geranyl diphosphate synthase
	evm.model.Contig10.559	GPPS2	Geranyl diphosphate synthase
	evm.model.Contig6.1490	GGPPS1	Geranylgeranyl diphosphate synthase
	evm.model.Contig47.210	GGPPS2	Geranylgeranyl diphosphate synthase
	evm.model.Contig6.1704	GGPPS3	Geranylgeranyl diphosphate synthase
	evm.model.Contig77.12	GGPPS4	Geranylgeranyl diphosphate synthase
