# Supplementary material for: The Chloranthus sessilifolius genome provides insight into early diversification of angiosperms
Source: Nat Commun. 2021 Nov 26;12:6929. doi: 10.1038/s41467-021-26931-3 (PMC8626421; doi:10.1038/s41467-021-26931-3)
Supplement: Supplementary file 3 — Reporting Summary [file 41467_2021_26931_MOESM3_ESM.pdf]

nature portfolio

Corresponding author(s): Yongzhi Yang

Last updated by author(s): Oct 21, 2021

Reporting Summary

Nature Portfolio wishes to improve the reproducibility of the work that we publish. This form provides structure for consistency and transparency in reporting. For further information on Nature Portfolio policies, see our Editorial Policies and the Editorial Policy Checklist.

Statistics

For all statistical analyses, confirm that the following items are present in the figure legend, table legend, main text, or Methods section.

n/a	Confirmed	
☐	☒	The exact sample size (*n*) for each experimental group/condition, given as a discrete number and unit of measurement
☐	☒	A statement on whether measurements were taken from distinct samples or whether the same sample was measured repeatedly
☐	☒	The statistical test(s) used AND whether they are one- or two-sided<br>*Only common tests should be described solely by name; describe more complex techniques in the Methods section.*
☐	☒	A description of all covariates tested
☐	☒	A description of any assumptions or corrections, such as tests of normality and adjustment for multiple comparisons
☐	☒	A full description of the statistical parameters including central tendency (e.g. means) or other basic estimates (e.g. regression coefficient) AND variation (e.g. standard deviation) or associated estimates of uncertainty (e.g. confidence intervals)
☐	☒	For null hypothesis testing, the test statistic (e.g. *F*, *t*, *r*) with confidence intervals, effect sizes, degrees of freedom and *P* value noted<br>*Give P values as exact values whenever suitable.*
☐	☒	For Bayesian analysis, information on the choice of priors and Markov chain Monte Carlo settings
☒	☐	For hierarchical and complex designs, identification of the appropriate level for tests and full reporting of outcomes
☐	☒	Estimates of effect sizes (e.g. Cohen's *d*, Pearson's *r*), indicating how they were calculated

Our web collection on statistics for biologists contains articles on many of the points above.

Software and code

Policy information about availability of computer code

Data collection	No software was used to collect the data
Data analysis	Genome size estimation: findGSE v1.94 and Jellyfish v2.3.0<br>Genome assembly: Nextdenovo v1.1.1, Smartdenovo v1.0, Nextpolish v1.2.0, Bowtie2 v2.3.0 and LACHESIS v1.0<br>Genome assessment: bwa v0.7.12-r1039, Trinity v2.8.4, BLAT v35 and BUSCO v3.0.<br>Genome annotation: Tandem Repeats Finder v4.04, RepeatMasker v4.0.7, RepeatModeler v1.0.11, LTR_retriever v2.8, LTRharvest v1.6.1, LTR_FINDER v1.0.7, r8s v1.81, BLASTP v2.3.0, PASA v2.3.3, AUGUSTUS v3.2.3, Genscan v1.0, GlimmerHMM v3.0.4, GeMoMa v1.6.1, EvidenceModeler v1.1.1 and InterProScan v5.36-75.0.<br>Polyploidization analysis: WGDI v0.4.1, PAML v4.9h, IQ-TREE v1.6.9 and ASTRAL v5.6.<br>Phylogenetic analyses: SonicParanoid v1.0, MAFFT v7.402, PAL2NAL v14, Phyx v1.1, IQ-TREE v1.6.9, ASTRAL v5.6.1, ASTRAL-pro vpaper, OrthoMCL v2.0.9, STAG v1.0.0, DensiTree v2.2.7, TreeShrink v1.3.3, GetOrganelle v1.7.4.1 and DiscoVista v1.0<br>Divergence time estimation: MCMCTree in the PAML package v4.9h.<br>Inferring ILS: Phybase v1.5, DendroPy v 3.12.0.<br>Inferring gene flow: PhyloNetworks v.0.9.0<br>Flower gene identify: BLASTP v2.3.0, HMMER v3.3, iTAK v1.7a, InterProScan v5.36-75.0.<br>Transcriptome analysis: Trinity v2.06, HISAT2 v2.2.1, StringTie v2.1.3<br>All the custom scripts and specific command lines have deposited in GitHub (https://github.com/yongzhiyang2012/Chloranthus-sessilifolius-genome).

For manuscripts utilizing custom algorithms or software that are central to the research but not yet described in published literature, software must be made available to editors and reviewers. We strongly encourage code deposition in a community repository (e.g. GitHub). See the Nature Portfolio guidelines for submitting code & software for further information.

Data

Policy information about availability of data

All manuscripts must include a data availability statement. This statement should provide the following information, where applicable:

- Accession codes, unique identifiers, or web links for publicly available datasets
- A description of any restrictions on data availability
- For clinical datasets or third party data, please ensure that the statement adheres to our policy

All of the raw sequence reads used in this study have been deposited at NCBI under the BioProject accession number PRJNA759285 (https://www.ncbi.nlm.nih.gov/bioproject/PRJNA759285). The assembly and annotation files are deposited in the Genome Warehouse in BIG Data Center (http://bigd.big.ac.cn) under the BioProject accession number PRJCA006913 (https://ngdc.cncb.ac.cn/bioproject/browse/PRJCA006913). Source data are provided with this paper. All other data are available from the corresponding author upon reasonable request.

Field-specific reporting

Please select the one below that is the best fit for your research. If you are not sure, read the appropriate sections before making your selection.

- [x] Life sciences
- [ ] Behavioural & social sciences
- [ ] Ecological, evolutionary & environmental sciences

For a reference copy of the document with all sections, see nature.com/documents/nr-reporting-summary-flat.pdf

Life sciences study design

All studies must disclose on these points even when the disclosure is negative.

Sample size	One mature Chloranthus sessilifolius individual was collected in the field and its fresh leaves snap-frozen in liquid nitrogen, then the frozen fresh samples were used to the genome sequencing. Three replications for different tissues (leaf, xylem, phloem, lobe, stamen and pistil) from the other three young Chloranthus sessilifolius individuals were also collected, and used for transcriptome sequencing.
Data exclusions	For the long reads, we filtered the low-quality (mean_qscore < 7) reads, and then further corrected by Nextdenovo (read_cutoff=2k, seed_cutoff=30k, blocksize=1.5g). For the Illumina short reads the following criteria were performed to filter the low quality reads: (i) containing more than 5% unidentified nucleotides, (ii) more than 65% of bases with a Phred quality score < 7, and (iii) more than 10 bp adapter sequences (allowing 2 bp mismatches)
Replication	All the tissues for RAN sequencing have three replications to make the transcriptome analysis more accuracy, and the high correlations were detected between replications.
Randomization	No randomization in our analysis. Within the genome assembly, annotation, comparison and whole genome duplication analyses, randomization is not needed. For the phylogeny analysis, all orthologous genes were used. For the hybridization and ILS analyses, all data (observed or simulated) were used, so no randomization needed.
Blinding	We collected the plant samples in the wild and sequenced and assembled the Chloranthus sessilifolius genome with no control group is referred here. Blinding is not applicable in this study.

Reporting for specific materials, systems and methods

We require information from authors about some types of materials, experimental systems and methods used in many studies. Here, indicate whether each material, system or method listed is relevant to your study. If you are not sure if a list item applies to your research, read the appropriate section before selecting a response.

Materials & experimental systems

n/a	Involved in the study	
[x]	[ ]	Antibodies
[x]	[ ]	Eukaryotic cell lines
[x]	[ ]	Palaeontology and archaeology
[x]	[ ]	Animals and other organisms
[x]	[ ]	Human research participants
[x]	[ ]	Clinical data
[x]	[ ]	Dual use research of concern

Methods

n/a	Involved in the study	
[x]	[ ]	ChIP-seq
[x]	[ ]	Flow cytometry
[x]	[ ]	MRI-based neuroimaging
